# Endoplasmic reticulum stress related super-enhancers suppress cuproptosis via glycolysis reprogramming in lung adenocarcinoma

**DOI:** 10.1038/s41419-025-07613-0

**Published:** 2025-04-19

**Authors:** Yan Gu, Hongchang Wang, Wentao Xue, Linjia Zhu, Chenghao Fu, Wenhao Zhang, Guang Mu, Yang Xia, Ke Wei, Jun Wang

**Affiliations:** 1 Department of Thoracic Surgery, Jiangsu Province Hospital and the First Affiliated Hospital of Nanjing Medical University, Nanjing, Jiangsu China; 2 Department of Anaesthesiology and Perioperative Medicine, Jiangsu Province Hospital and the First Affiliated Hospital of Nanjing Medical University, Nanjing, Jiangsu China; 3 Department of Thoracic Surgery, Taihe Hospital, Shiyan, Hubei China

**Keywords:** Cell death, Lung cancer

## Abstract

The role of copper in tumor progression is thought to be a double-edged sword. Moderate levels of copper promote tumor progression, while excess copper induces a novel form of programmed cell death known as cuproptosis. However, the relationship between lung adenocarcinoma (LUAD) and cuproptosis remains poorly understood. Copper colorimetric assay identified the progression of LUAD simultaneous associated with higher copper accumulation. Single-cell RNA sequencing further identified the activation of unfolded protein response correlates with copper accumulation, particularly the spliced form of XBP1 (XBP1s). XBP1s negatively regulates the protein level of LIPT1 to inhibit LUAD cell death induced by copper-loaded ionophore elesclomol. CUT&Tag-seq and chromosome conformation capture (3 C) experiment showed that XBP1s affect the frequency of MGRN1 promoter-enhancer interactions in various copper environments by forming super-enhancers. Additionally, MGRN1 promotes the ubiquitination and degradation of LIPT1, which in turn supports glycolysis in LUAD cells. In mouse xenograft models, overexpression of XBP1s significantly inhibits the cuproptosis induced by copper ionophores. Co-administration with SEs inhibitor and copper ionophore also markedly reduced tumor volume and growth rate. Our study sheds light on the molecular mechanism by which XBP1s affect the cuproptosis through super-enhancers formation in LUAD and suggested the potential clinical value of copper ionophore as well as a potential biomarker XBP1s for treatment response.

## Introduction

Lung cancer remains the leading cause of cancer-related deaths worldwide, with lung adenocarcinoma (LUAD) being the predominant histological subtype [1, 2]. Despite considerable advances in research on LUAD, it remains to be one of the most prevalent and lethal malignancies, characterized by histologic heterogeneity as well as molecular heterogeneities [3]. Homeostasis of copper metabolism is crucial for maintaining tumor cell viability and cancer progression [4]. In 2022, Tsvetkov et al. discovered and characterized a novel form of cell death called cuproptosis, which is distinct from other forms of programmed cell death due to its dependence on excess copper ions [5]. Cuproptosis occurs when copper directly binds to lipoylated components of the tricarboxylic acid cycle, leading to aggregation of lipoylated proteins and loss of iron-sulfur cluster proteins. This induces proteotoxic stress, culminating in cell death. This newly discovered cell death mechanism may present a promising target for LUAD treatment. However, the role of cuproptosis in LUAD remains largely unexplored.

Liquid-liquid phase separation (LLPS) is a novel principle for explaining the precise spatial and temporal regulation in tumor cells [6]. LLPS compartmentalizes proteins and nucleic acids into liquid-like bodies with specific functions, which have recently been termed biomolecular condensates [7, 8]. Super-enhancers (SEs) are large clusters of transcriptional enhancers that are distinguished from classical enhancers by extreme enrichment of transcriptional elements such as BRD4 and MED1. SEs are defined as clusters of enhancers located in close genomic proximity that regulate genes responsible for specifying cell identity and are functionally associated with cell type-specific biological processes. In contrast, classical enhancers and the genes they regulate are common across various cell types. Some cell type-specific SEs overlap with other previously identified regulatory regions implicated in tissue-specific gene expression, such as locus control regions, DNA methylation valleys, and transcription initiation platforms. This suggests that these diverse regulatory elements may represent distinct classes of SEs. [9, 10]. Moreover, SEs are thought to be a product of LLPS in the nucleus by TF, coactivators, and mediators enriched in intrinsic disordered regions (IDRs) [11]. During malignant transformation, deregulated SEs promote oncogenic signaling pathways and processes and thus serve as key regulators of cancer development as well as promising therapeutic targets [12, 13]. In short, SEs play a critical role in cancer by maintaining cancer cell identity, regulating key oncogenes, and conferring tumor dependencies. They are present in various cancer types and can be targeted by inhibiting chromatin and transcriptional regulators. However, key questions remain about the contributions of individual SE components to cancer phenotypes and their response to therapy. Establishing the relevance of SE-driven oncogenic dependencies in primary tumor models will be essential for identifying new therapeutic vulnerabilities.

The endoplasmic reticulum (ER) stress response or unfolded protein response (UPR) orchestrates adaptive programs to promote cancer cell survival [14, 15]. It has been reported IRE1α–XBP1 is the most evolutionarily conserved arm of the UPR. Upon cellular stress caused by misfolded or unfolded proteins, the UPR is activated and the RNase activity of IRE1 promotes the splicing of XBP1u mRNA, which generates the stress-responsive transcription factor XBP1s [16]. XBP1s then translocate to the nucleus, where it promotes the transcription of genes that mitigate ER stress [17]. XBP1s has been implicated as an oncogene in lung cancer that promote tumor cell adaptation to extreme tumor microenvironments such as hypoxia, nutrient deprivation, and oxidative stress, all of which are commonly encountered in solid tumors. In these situations, XBP1s helps tumor cells by promoting the expression of genes that enhance cell survival, protein folding, and stress response pathways, thereby enabling tumor cells to cope with these adverse conditions [18–20]. However, whether XBP1s can help LUAD cells resist copper-induced death remains unclear.

## Materials/Subjects and Methods

### Patients and sample collection

The LUAD tissue microarrays (TMAs) containing 80 cases of LUAD tissue specimens, purchased from Changsha Yaxiang Biotechnology, were used for XBP1s, DLAT and FDX1 staining. The study using the tissue microarray was approved by the Life Sciences Ethics Committee of Changsha Yaxiang Biotechnology Co., LTD (Approval number: Csyayj2024034).

Fresh LUAD tissue samples were collected from the thoracic surgery department of Jiangsu province hospital. This study was approved by the Ethics Committee of Jiangsu Provincial People’s Hospital (Approval Number: 2024-SR-139). Informed consent was obtained from all participants prior to the collection of human tissue samples.

### Singe-cell sequencing data analysis

Surgical specimens for single-cell sequencing were obtained from the Department of Thoracic Surgery, Jiangsu Province Hospital, and the First Affiliated Hospital of Nanjing Medical University. Freshly collected tissue samples were washed twice in pre-chilled RPMI 1640 medium containing 0.04% BSA under sterile conditions. The tissue was minced into approximately 0.5 mm³ fragments using surgical scissors and digested in an enzyme cocktail at 37 °C for 30–60 min with intermittent gentle mixing every 5–10 min. The digested suspension was filtered through a 40 μM cell strainer one to two times, centrifuged at 300 g for 5 min at 4 °C, and resuspended in an appropriate volume of culture medium.

Red blood cells were lysed using red blood cell lysis buffer (MACS, German, #130-094-183) for 10 min at 4 °C, followed by centrifugation at 300 g for 5 min. After washing once with culture medium, the cell pellet was resuspended in 100 μL of medium. Cell concentration and viability were assessed using the Luna cell counter. Dead cells were removed using the MACS Dead Cell Removal Kit (German, #130-090-101) according to the manufacturer’s instructions. For single-cell RNA sequencing, the single-cell suspension was adjusted to a concentration of 700–1200 cells/μL. Library preparation was performed using the 10x Genomics Chromium Next GEM Single Cell 3ʹ Reagent Kits v3.1 (1000268) following the manufacturer’s protocol. Libraries were sequenced using the Illumina NovaSeq 6000 platform with PE150 high-throughput sequencing.

Raw sequencing data were processed using the Cell Ranger pipeline (10x Genomics) to generate unique molecular identifier (UMI) count matrices. Quality control and downstream analysis were conducted using the R package Seurat (version 4.0.3). UMI count matrices were converted into Seurat objects, and the main cell clusters were identified using the FindClusters function with a resolution of 0.1. Clusters were visualized using 2D t-SNE plots. Differentially expressed genes (DEGs) between low- and high-grade derived cells were identified using the FindAllMarkers function in Seurat. Gene Ontology (GO) and KEGG enrichment analyses of DEGs were performed using the clusterProfiler package (version 3.18.1).

### Cell lines, cell culture, plasmids, transfection, lentiviral production, and infection

Human LUAD A549, H2087, PC-9, and H1975 cell lines and human embryonic kidney HEK293T cell lines were obtained from the ATCC. A549 and HEK293T cell lines were cultured in DMEM and 10% FBS. H2087, PC-9, and H1975 cell lines were cultured in RPMI-1640 and 5% FBS.

Plasmids, siRNAs, lentiviral expression vectors, negative controls, XBP1s, LIPT1, and XBP1s-shRNA were designed and synthesized by Hanbio (Shanghai, China). The cells were transfected using Lipofectamine 3000 (Thermo Fisher Scientific, #L3000015, USA) according to the manufacturer’s instructions.

For lentivirus construction, oligonucleotides with the following targeting sequences were used to clone small hairpin RNA (shRNA)-encoding sequences into the hU6-MCS-PGK-EGFP lentiviral RNAi vector (Hanbio, Shanghai, China): XBP1, 5’-AAAAAATTTTTTTAAAAAA-3’. The recombinant lentivirus with XBP1 was produced by co-transfection of 293T cells with plasmids PSPAX2 and PMD2G using LipoFiter (Hanbio, Shanghai, China). Lentivirus-containing supernatant was harvested 48 h after transfection and filtered through 0.22 μm cellulose acetate filters (Millipore, USA). The recombinant lentiviruses were concentrated by ultracentrifugation (2 h at 50,000 × g). To establish stable XBP1-knockdown cell lines, A549 and PC9 cells were transfected with lentiviral RNAi vector at an MOI of approximately 10 in the presence of 5 μg/mL polybrene. After 24 h, the culture medium was removed and fresh medium was added. For stable cell line selection, 5 mg/mL puromycin was added to the medium 72 h after transduction. Empty lentivector lenti-puromycin was used as a negative control. After harvesting the cells, the expression level of XBP1 was determined by real-time PCR.

For the construction of XBP1s, LIPT1, and vector overexpression lentivirus, cDNA cloned by PCR was inserted into CMV-MCS-IRES-puromycin lentiviral vectors (Hanbio, Shanghai, China). The recombinant lentivirus with a gene-coding sequence was produced by co-transfection of 293 T cells with the plasmids PSPAX2 and PMD2G using LipoFiter^TM^ (Hanbio, Shanghai, China). shRNA cell lines were constructed using a similar procedure.

### Antibodies

Antibodies used in this study include: XBP1s (Cell Signaling Technology, #40435, 1:1000 for WB, 1:300 for IF, 1:50 for CUT&Tag); XBP1s (Merck, #MABC521, 1:400 for IHC); DLAT (Proteintech, # 68303-1-Ig, 1:400 for IHC and 1:1000 for WB); FDX1 (Proteintech, # 12592-1-AP, 1:400 for IHC); P300 (Cell Signaling Technology, #D2X6N, 1:300 for IF, 1:50 for CUT&Tag); MED1 (Thermo Fisher, #PA5-36114, 1:300 for IF, 1:50 for CUT&Tag); BRD4 (Cell Signaling Technology, #13440, 1:300 for IF, 1:50 for CUT&Tag); RNA Pol II-S2P (Millipore, cat# 04-1571, 1:300 for IF); RNA Pol II-S5P (Millipore, cat# 04-1572, 1:300 for IF); H3K4me3 (Cell Signaling Technology, cat# 9751, 1:300 for IF); H3K27ac (PTMbio, #PTM-160, 1:300 for IF, 1:50 for CUT&Tag); H3K4me1 (PTMbio, #PTM-5158, 1:300 for IF); MGRN1 (Proteintech, #11285-1-AP, 1:2000 for WB, 1:200 for IHC); β-actin (Proteintech, # 81115-1-RR, 1:10000 for WB); LIPT1 (Thermo Fisher, #PA5-106991, 1:1000 for WB and 1:200 for IHC); anti-DYKDDDDK tag (Proteintech, # 80010-1-RR, 1:5000 for WB); anti-HA tag (Proteintech, # 81290-1-RR, 1:5000 for WB); anti-His tag (Proteintech, #66005-1-Ig, 1:5000 for WB); HRP-conjugated goat anti-rabbit (Proteintech, # SA00001-2, 1:10000 for WB); HRP-conjugated goat anti-mouse (Proteintech, # SA00001-1, 1:10000 for WB); CoraLite® Plus 488-Goat Anti-Mouse Recombinant Secondary Antibody (H + L) (Proteintech, # RGAM002, 1:1000 for IF); CoraLite® Plus 594-Goat Anti-Mouse Recombinant Secondary Antibody (H + L) (Proteintech, # RGAM004, 1:1000 for IF); CoraLite® Plus 647-Goat Anti-Mouse Recombinant Secondary Antibody (H + L) (Proteintech, # RGAM005, 1:1000 for IF)

### Tissue copper (Cu) colorimetric assay and intracellular copper probe staining

Cu levels in LUAD tissues were detected using a Cu colorimetric assay kit (# E-BC-K300-M; Elabscience, Wuhan, China). The Cu concentration was determined from the absorbance at 580 nm using a standard curve.

Copper fluorescence probes were purchased from Targetmol (#T40996, USA). LUAD cells were incubated into confocal dishes 24 h in advance. Then we incubated cells with complete medium containing 100 μM CuCl_2_ for 10 h. After the medium change, 5 μM Bathocuproine disulfonate disodium (#HY-W034953; MedChemExpress, China) was added for 20 min to chelate the Cu extracellularly. After another complete culture medium exchange, the medium containing 10 μM copper probe was added for 30 min to label the copper in cells with fluorescence. Finally, a Stellaris STED confocal microscope (Leica, Germany) was used to determine fluorescence intensity.

### Cell viability assay and EdU assay

Relative cell numbers following treatment with elesclomol (#HY-12040; MedChemExpress, China) were determined by plating cells at 10,000 cells/well in 96 well plates. Indicated concentrations of ES and 1 μM CuCl_2_ were added 16–24 h after plating at least in triplicate for each condition. The culture medium was removed and refreshed after pulse treatment for 6 h. Cell viability was measured 72 h after drug addition using a Luminescent Cell Viability Assay (#G7573, Promega, USA) according to the manufacturer’s protocol, and luminescence was measured using a multimode microplate reader (Biotek, Synergy H1, USA).

The EdU assay was performed to determine the cell proliferation capacity of A549 and PC9 cells treated with ES. The kFluor647-EdU kit was purchased from KeyGen Biotech (#KGA9604-100; China). The images were captured using a THUNDER DMi8 fluorescence microscope (Leica).

### 4D-DIA proteomic analysis

XBP1s-OE and expression vector A549 cells pulse treated with 40 nM of ES (with 1 µM CuCl_2_) for 2 h were collected for protein analysis 24 h after treatment. Reaction solution (1% SDC, 10 mM TCEP, 40 mM CAA) was added to the samples and incubated at 60 °C for 30 min for protein denaturation, disulfide bond reduction, and sulfhydryl alkylation. The Bradford method was used to determine protein concentration. The SDC concentration was diluted to less than 0.5%, trypsin was added at a 1:50 enzyme-to-protein mass ratio, and incubated at 37 °C with shaking overnight for digestion. The next day, TFA was added to terminate the digestion, and the pH of the solution was adjusted to approximately 6.0. The solution was centrifuged at 12,000 × g for 15 min and the supernatant was desalted. After desalting, the peptide solution was dried by centrifugation concentrator, and then frozen at −20 °C for on-line testing.

Mass spectrometry (MS) was performed using DIA with an Ultimate 3000 (capillary flow) coupled with a Q Exactive HFX system. The prepared peptide samples were firstly bound to a Trap column and then separated on an analytical column (300 μm × 150 mm, 2 μm particle size, 100 Å pore size, Acclaim PepMap C18 column, Thermo). The two mobile phases used to establish the analytical gradient were buffer A-0.1% (V/V) formic acid, 2% ACN, 3% ACN, 2% ACN, 3% ACN, 3% ACN, 3% ACN, and 3% ACN. (v/v) formic acid, 2% ACN, 3% DMSO in H2O, buffer B: 0.1% (v/v) formic acid and 3% DMSO in acetonitrile. The mass spectra files obtained from DIA scanning were processed using DIA-Umpire to obtain secondary mass spectra files that could be used for database search. The database search of the secondary mass spectra was performed using the software MSFragger, and the results obtained were used as a spectrum library for subsequent target extraction. The algorithm used for DIA target extraction quantification was DIA-NN. The detection results were screened with 1% FDR. The protein quantification intensity information obtained from the DIA analysis was normalized by log2 transformation, data padding, and data normalization techniques. t-test analysis was used for difference comparisons. Differential proteins were screened using differential fold ratios and p-values.

### Immunohistochemistry and immunofluorescence

Tissue samples were collected and fixed in 10% formalin for 24 h. Fixed tissues were embedded in paraffin and sectioned at 4 µm thickness using a microtome. Tissue sections were deparaffinized in xylene for 10 min (three times) and rehydrated using a graded ethanol series (100%, 95%, 70%) followed by rinsing with distilled water. The slides were then immersed in a citrate buffer and heated in a microwave oven for 10 min. They were allowed to cool for 20 min in the buffer at room temperature. Tissue sections were treated with 3% hydrogen peroxide in methanol for 10 min to quench endogenous peroxidase activity, followed by rinsing with distilled water. Sections were incubated overnight at 4 °C with the primary antibodies. The slides were washed in PBS (three times for 5 min each). Finally, the sections were incubated with biotinylated secondary antibodies for 30 min at room temperature and washed with PBS.

The cells were inoculated onto coverslips and set aside for 16–24 h prior to immunofluorescence staining. After adhesion, cells were washed, fixed with 4% paraformaldehyde for 30 min, permeabilized with 0.5% Triton X-100 for 20 min, and blocked with 2% BSA for 1 h. Cells were incubated in the diluted antibody for 1 h at room temperature or overnight at 4 °C. The cells were washed three times in PBS and incubated with secondary antibody for 1 h at room temperature in the dark. The fluorescence localization of the target proteins was observed using confocal microscopy.

### Transmission Electron Microscopy (TEM)

Transmission Electron Microscopy (TEM) was utilized to analyze the samples in this study. The sample preparation involved the following steps: The material was initially sectioned into thin slices approximately 100 nanometers thick. These slices were then further thinned to around 50 nanometers using an ion milling technique to ensure adequate electron transparency. The thinned samples were cleaned using ultrasonic treatment to remove surface contaminants and were subsequently dried in a clean environment.

TEM observations were conducted using a Hitachi HT7700 transmission electron microscope (Tokyo, Japan), operating at an accelerating voltage of 80.0 kV. The samples were supported on copper grids with a thin carbon film to improve sample stability. High-resolution imaging mode was employed to capture atomic-level details of the samples. Images were collected with a Gatan Orius CCD camera, and image processing and analysis were performed using DigitalMicrograph software.

### Protein expression and purification

Transformed colonies were screened for protein expression by inoculating single colonies into 5 mL LB medium containing 50 µg/mL kanamycin, followed by overnight incubation at 37 °C with shaking at 200 rpm. An overnight culture was used to inoculate 500 mL of LB medium at an OD600 of 0.1. The culture was incubated at 37 °C until the OD600 reached 0.6–0.8, at which point protein expression was induced by adding IPTG to a final concentration of 0.5 mM. Bacteria were harvested by centrifugation at 6000 × g for 15 min and resuspended in lysis buffer (20 mM HEPES, 0.2 mM EDTA, 100 mM KCl, 20% glycerol, 1% Triton, 2 mM PMSF, and 1 mg/mL lysozyme). The lysate was centrifuged at 12,000 × g for 30 min at 4 °C. The supernatant was applied to a Ni-NTA agarose column pre-equilibrated with lysis buffer. The recombinant proteins were eluted using an elution buffer. Fractions containing target proteins were identified by SDS-PAGE and pooled.

### Droplet assay

Recombinant EGFP- or mCherry-fusion proteins were diluted to appropriate concentrations using 50 mM Tris-HCl (pH 7.4). Recombinant proteins were added to solutions containing 125 mM NaCl and 10% PEG8000 as a crowding agents. The protein solution was then immediately loaded onto a glass slide and covered with a coverslip. The slides were imaged using a Stellaris STED confocal microscope (×63, Leica, Germany).

### Fluorescence recovery after photo-bleaching (FRAP)

FRAP was performed using a Leica Stellaris STED confocal microscope with a 63× oil-immersion objective. Images were acquired using LASX software. After selecting suitable protein droplets and recording three frames prior to the bleach pulse, bleaching was performed using a 488 nm argon laser at a fitting power of three frames. The fluorescence recovery was recorded every 2 s after bleaching.

### Co-Immunoprecipitation (Co-IP)

Cells were lysed in IP lysis buffer (Thermo Fisher Scientific, #87787, USA) supplemented with 1x HALT protease inhibitor (Thermo Fisher Scientific, #87785, USA) for 30 min on ice. Protein solutions were collected by centrifugation for 10 min at 10,000 RPM. Supernatant was incubated at 4 °C with the appropriate antibody overnight to form antigen-antibody complex. The next day, the target protein was adsorbed using protein A/G magnetic beads (Vazyme, #PB101-01, China), and immunoblot analysis was carried out. During IP experiments involving ubiquitination, MG132 was added 4 h in advance to prevent the degradation of ubiquitinated proteins.

### Western blot (WB) analysis

The cells were lysed in RIPA lysis buffer (Thermo Fisher Scientific, #89901, USA) supplemented with 1x HALT protease inhibitor (Thermo Fisher Scientific, #87785, USA) for 30 min on ice. Cell debris were removed by centrifugation for 15 min at 13,000 RPM. The protein abundance was quantified using the bicinchoninic acid (BCA) method. Size fractionation was performed using Bis-Tris 4–12% gels for SDS-PAGE, and the fractions were transferred onto PVDF membrane (Merck, #05317, Germany). The membrane was then incubated at room temperature for 20 min in quick blocking buffer and incubated at 4 °C with the appropriate antibody overnight.

### RNA extraction and reverse transcription and quantitative PCR (RT-qPCR)

Total RNA was isolated from cells using the FastPure Complex Tissue/Cell Total RNA Isolation Kit (Vazyme, #RC113-01, China) and reverse transcribed into cDNA using HiScript II Q RT SuperMix for qPCR (Vazyme, #R223-01, China), according to the manufacturer’s protocol. The primers used for the RT-qPCR are listed in an additional file.

### Cleavage Under Targets and Tagmentation (CUT&Tag)-seq and CUT&Tag-qPCR

CUT&Tag-seq was performed using the Hyperactive Universal CUT&Tag Assay Kit for Illumina Pro (#TD904-01; Vazyme, China). DNA fragmentation and extraction, library amplification, and PCR product purification were performed according to manufacturer’s instructions. All the primary antibodies used were of Chip or CUT&T AG grade. The primer sequences are listed in an additional file.

### Luciferase reporter assay

The reporter plasmids were constructed by HanBio (Shanghai, China). Cells in 24-well plates were co-transfected with the target and control plasmids. Gluc activity was measured 48 h after transfection.

### Chromosome conformation capture (3 C) experiment

Chromosome conformation capture kits were purchased from Bersin Bio (Guangzhou, China). Total of 1 × 10^7^ A549 or PC9 cells were cross-linked with 2% formaldehyde in 10% (v/v) FBS/PBS for 10 min at room temperature. The manufacturer’s instructions were followed for complete digestion, ligation, DNA extraction, and PCR amplification. Finally, the products were subjected to first-generation sequencing to confirm the presence of interactions between transcription elements. The primer sequences used in the 3 C assay are provided in an additional file.

### Quantification of lactate and pyruvate

L-lactate and pyruvate levels were measured in vitro using a L-lactate assay kit (# E-BC-K044-M, Elabscience, Wuhan, China), and a pyruvate assay kit (#ab65342, Abcam, UK).

### Measurement of OCR and ECAR

The extracellular acidification rate (ECAR) and oxygen consumption rate (OCR) of cells were assessed using a Seahorse XFe24 Flux Analyzer (Seahorse Bioscience, Agilent). A glycolytic stress test kit (Seahorse Cat. #103020–100) and a mitochondrial stress test kit (Seahorse Cat. #103015–100) were used to determine ECAR and OCR.

### Mouse xenograft study

Four-week-old male/female thymic BALB/c nude mice (15–20 g) were purchased from the Model Animal Research Center of Nanjing University (Nanjing, China) and placed in a temperature-controlled room with a 12 h light/dark cycle. Food and water were provided ad libitum. The A549 cells in a final volume of 100 μL were subcutaneously inoculated into the right flank of mice. The ES group was injected with ES 50 mg/kg every three days and the other group was injected with saline as a control. Tumor size was measured twice a week using a Vernier caliper, and tumor volume (V) was calculated using the following formula: V= length × (width^2^)/2. Five weeks after inoculation, the mice were humanely euthanized and all tumor xenografts were collected, photographed, and analyzed by immunofluorescence staining.

### Statistical analysis

All data were obtained from at least three independent experiments and are presented as means with either standard deviation (SD) or standard error of the mean (SEM), as indicated in the figure legends. Statistical analyses were performed using GraphPad Prism. For comparisons between two independent groups, a two-tailed unpaired Student’s t-test was applied. For paired data, a two-tailed paired Student’s t-test was used. To compare more than two groups, one-way or two-way analysis of variance (ANOVA) was employed, followed by appropriate post-hoc tests to assess differences between groups. The criteria for statistical significance are indicated as follows: *P < 0.05, **P < 0.01, ***P < 0.001 and ****P < 0.0001.

## Results

### Progression of LUAD is concomitant with accumulation of copper

**Fig. 1:**
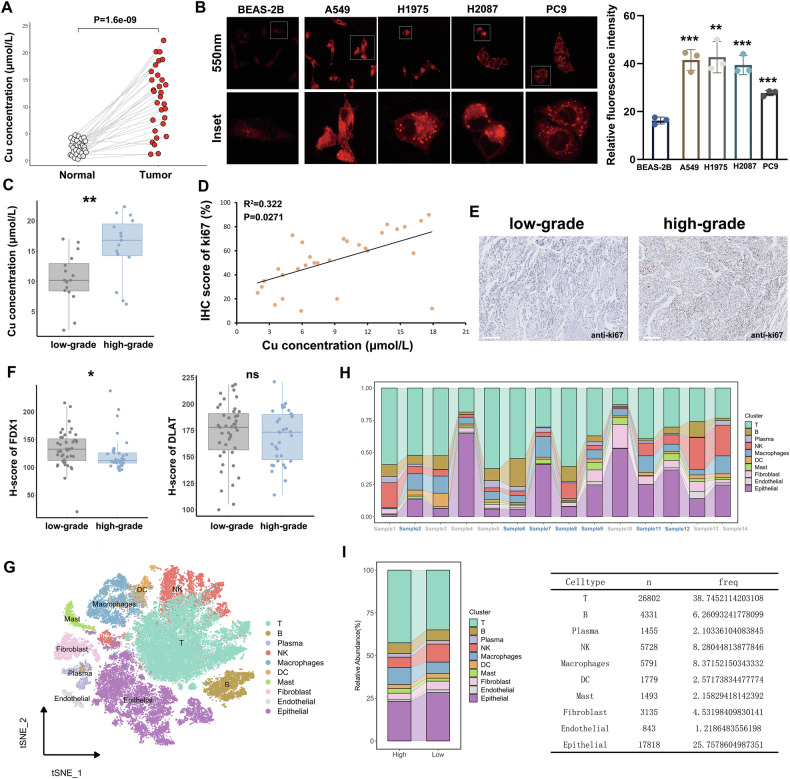
Pathological progression of LUAD is accompanied by the accumulation of copper. **A** Copper levels in LUAD tumor tissues (*n* = 30) were significantly higher than in matched normal tissues (*n* = 30), as determined by a colorimetric copper assay. Absorbance at 580 nm was measured. **B** Confocal fluorescence imaging revealed increased copper fluorescence (500 nm) in LUAD cell lines (A549, PC9, H2087, and H1975) compared to normal alveolar epithelial cells (BEAS-2B) following a 10 h incubation with 10 µM CuCl_2_. Fluorescence intensity was quantified and normalized to cell area (*n* = 3). **C** Box plot illustrating the relative copper (Cu) concentrations in fresh LUAD tissues, showing significantly higher copper levels in the high-grade group (*n* = 15) compared to the low-grade group (*n* = 15). **D** Pearson correlation analysis revealed a positive correlation between Ki-67 expression and copper (Cu) concentration (*n* = 30, R = 0.322, *p* = 0.0271). A simple linear regression line is included to illustrate the relationship. **E** Representative IHC images of Ki-67 staining in low- and high-grade LUAD tissues. **F** Box plot illustrates the distribution of H-scores for DLAT and FDX1 expression levels, as determined by immunofluorescence staining on a LUAD tissue microarray (*n* = 80). The H-score was calculated as: 1×(percentage of cells with staining score 1) + 2×(percentage of cells with staining score 2) + 3×(percentage of cells with staining score 3). The results show that FDX1 expression is significantly higher in the low-grade group compared to the high-grade group, while no significant difference is observed for DLAT expression. **G** t-SNE clustering analysis of single-cell RNA sequencing data reveals ten distinct cellular subpopulations. The frequency distribution of different cellular subpopulations in each LUAD sample **H** and the comparison of subpopulation frequencies between low- and high-grade LUAD groups (**I**). The data are presented as the mean ± SEM. *p < 0.05, **p < 0.01, ***p < 0.001, ****p < 0.0001.

We selected surgical specimens from 30 patients with LUAD to assess the copper (Cu) content in tumor and normal tissues using a colorimetric assay. The results showed that the Cu concentration in tumor tissue was significantly higher than in normal tissue (Fig. 1A). To further explore this, we performed confocal imaging on LUAD cell lines and observed that LUAD cells took up more Cu compared to normal lung epithelial cells under the same conditions (Fig. 1B). These findings indicate that LUAD tumor tissue and cells exhibit elevated Cu uptake, suggesting a potential association between Cu and LUAD, but further studies are needed to clarify the role of Cu in LUAD progression.

By testing the Cu content of tissues, we found that Cu levels varied between tumor tissues. Poorly differentiated tissues tend to contain higher levels of Cu. According to the 2021 WHO classification of lung tumors, we defined grade 1/2 invasive non-mucinous LUAD as low-grade and grade 3 invasive non-mucinous LUAD as high-grade. By examining 15 fresh samples each from low- and high-grade tumors, we found that the high-grade tumor tissues contained higher levels of Cu (Fig. 1C). Furthermore, Cu content was positively correlated with Ki-67 expression, a marker of cell proliferation (Fig. 1D, E). During cuproptosis, mitochondrial copper can directly bind to lipoylated proteins, leading to the aggregation of these proteins, especially dihydrolipoamide S-acetyltransferase (DLAT). In addition, ferredoxin 1 (FDX1) is implicated in the upregulation of protein lipoylation during cuproptosis. We used tissue microarrays to detect the expression of DLAT and FDX1 in the different pathological grade LUAD tissues (Fig. S1A). The results demonstrate that FDX1 expression is significantly reduced in the high-grade group compared to the low-grade group. Although DLAT shows a similar trend, this difference does not reach statistical significance (Fig. 1F). In summary, LUAD progression is associated with copper accumulation, but elevated cuproptosis levels were not observed.

### XBP1s rescues LUAD cells from copper ionophore-mediated cell death

**Fig. 2:**
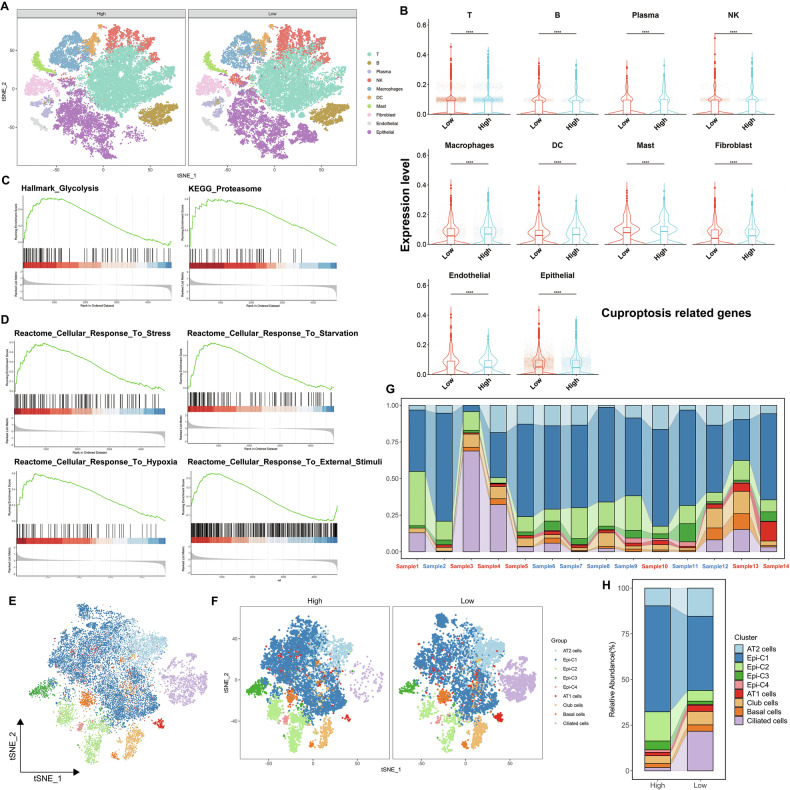
Characterization of cellular heterogeneity, cuproptosis-related gene expression, and pathway enrichment in low- and high-grade LUAD. **A** t-SNE plots of single-cell transcriptomes derived from tumors classified as high-grade (left) or low-grade (right). Each point represents an individual cell, colored by its assigned cell type. **B** Violin plots comparing cuproptosis-related gene expression levels across the indicated cell populations in low-grade versus high-grade tumors. Significance levels are denoted (***p < 0.001). **C**, **D** Gene Set Enrichment Analysis (GSEA) of high-grade vs. low-grade groups, showing enrichment profiles for metabolic (Glycolysis and Proteasome) and stress-associated (Cellular response to stress, Cellular response to starvation, Cellular response to hypoxia and Cellular response to external stimuli) pathways. Black vertical lines in the enrichment plots indicate leading edge genes contributing to the enrichment signals. **E** Second-level t-SNE clustering of epithelial cells, revealing distinct subpopulations within the epithelial compartment. **F** t-SNE visualization comparing the distribution of epithelial subclusters in high-grade (left) and low-grade (right) tumors. Stacked bar plots showing the proportion of major cell types across individual tumor samples (**G**) and high- vs. low-grade groups (**H**).

To investigate the molecular mechanisms underlying this paradoxical phenomenon in LUAD, we selected seven low-grade and seven high-grade specimens for single-cell sequencing analysis. After quality control, data normalization, and Principal Component Analysis (PCA), the cells from the LUAD samples were categorized into 10 cell types using the t-distributed stochastic neighbor embedding (t-SNE) algorithms (Fig. 1G). TOP5 marker genes regarding these cell types are shown in Fig. S1B. We examined the frequency variations of different cell populations across samples and compared cell population frequencies between the low- and high-grade tumor groups (Fig. 1H, I). Figure 2A shows the t-SNE analysis of cell cluster distribution across low- and high-grade groups.

Based on the previous study, we focused on assessing the expression of cuproptosis-related genes (FDX1, LIPT1, DLAT, PDHA1, PDHB, DLD, LIAS and DLST) in different cell subpopulations [6]. Our analysis revealed that in most cell populations, the expression of these genes was significantly higher in the low-grade group, with the most pronounced difference observed in epithelial cells, consistent with our earlier tissue microarray staining results (Fig. 2B). We then performed Gene Set Enrichment Analysis (GSEA) using well-established databases: Kyoto Encyclopedia of Genes and Genomes (KEGG), Gene Ontology (GO), Hallmark and Reactome. In the high-grade group, GSEA revealed significant enrichment in pathways related to glycolysis and proteasome (Fig. 2C), suggesting alterations in energy metabolism and protein degradation. Importantly, we observed significant enrichment in pathways related to cellular stress responses, particularly those associated with the ER stress response (Fig. 2D). This finding prompted us to hypothesize that ER stress may link to cuproptosis. Additionally, we identified that pathways related to protein localization to the ER and metal ion homeostasis were significantly enriched in the high-grade group (Fig. S1C). Given that previous studies have established a link between ATF4 in the UPR pathway and cuproptosis [21], we hypothesize that another arm of the UPR pathway, the IRE1α-XBP1 signaling pathway, may also be involved in cuproptosis.

To further investigate the heterogeneity of epithelial cells, we performed secondary clustering analysis using t-SNE. The epithelial cells were refined into nine distinct subpopulations, including four tumor cell clusters (Epi-C1, Epi-C2, Epi-C3, Epi-C4) and five non-tumor cell clusters (Fig. 2E, F). The top 5 marker genes for each cell population are presented in Fig. S1E, and the frequency variations across samples and groups are shown in Fig. 2G, H. Similar to the previous analysis, the cuproptosis gene set showed higher expression in the refined tumor cell clusters of the low-grade group, except for the Epi-C2 cluster (Fig. S1D). Additionally, GSEA performed on the Epi-C1 cluster, which had the largest cell population, revealed significant enrichment of glycolysis and cellular stress response pathways in the high-grade group (Fig. S1F). Overall, the results of the secondary clustering analysis were consistent with those observed in the total epithelial cell population.

**Fig. 3:**
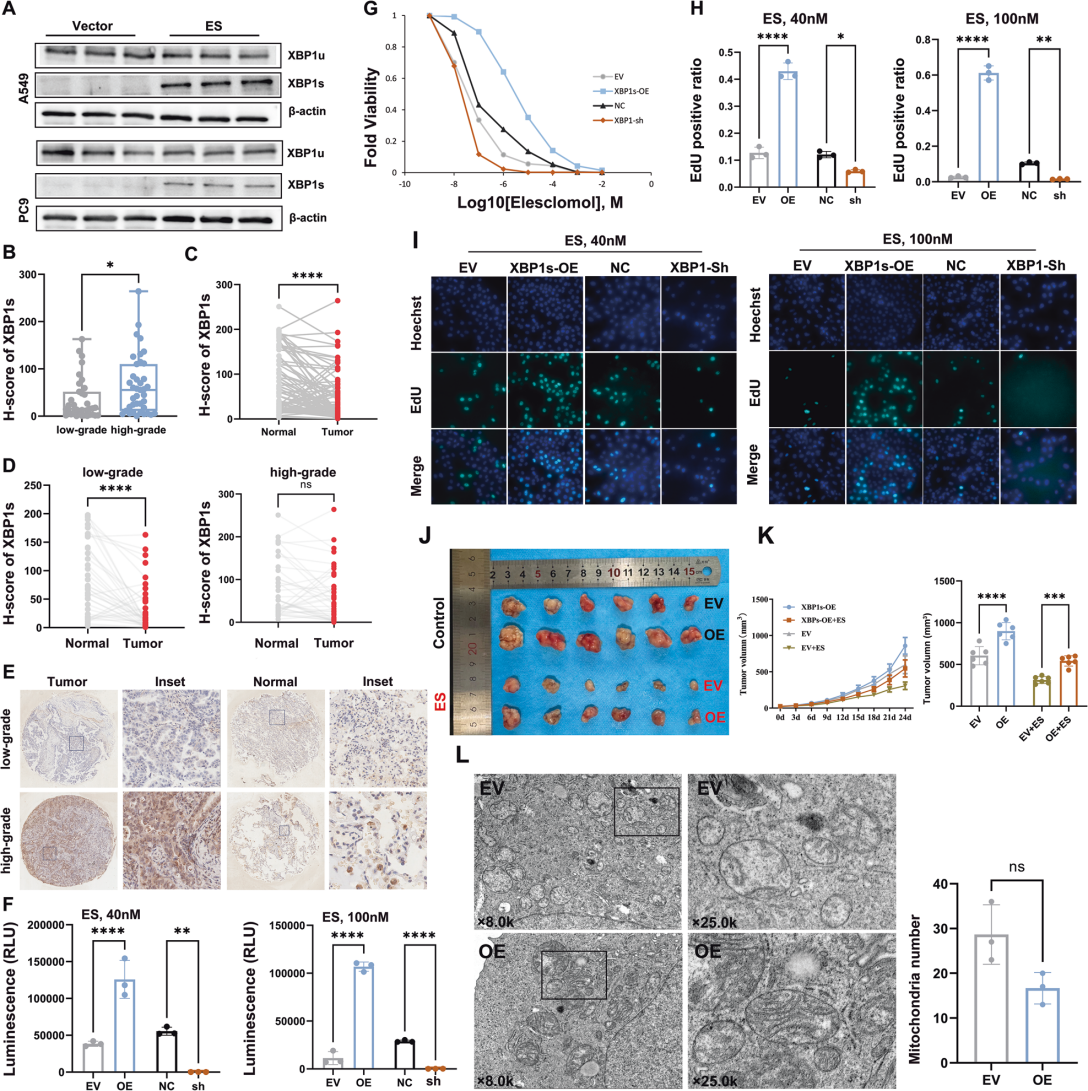
XBP1s protects LUAD cells from cell death induced by ES treatment. **A** WB analysis confirmed a significant upregulation of XBP1s expression in LUAD cell lines A549 and PC9 following ES treatment. **B** Box plot illustrates the distribution of H-scores for XBP1s expression in tumor tissue microarray from low- (*n* = 38) and high-grade (*n* = 39) LUAD. The H-scores in the high-grade group are significantly higher than those in the low-grade group. **C** Box plot illustrates the paired H-scores of XBP1s expression in tumor and adjacent normal tissues from a LUAD tissue microarray (*n* = 77), with statistical analysis performed using a paired t-test. **D** H-scores of XBP1s expression in tumor and adjacent normal tissues from the tissue microarray, stratified into low- (*n* = 38) and high-grade (*n* = 39) groups. Statistical analysis was performed using a paired t-test within each group. **E** Representative IHC images of XBP1s staining in low- and high-grade tissues. **F** After treating A549 cells with 40 nM and 100 nM ES for 72 hours, cell viability was measured. In the XBP1-overexpression (OE) group, viability was significantly higher compared to the empty vector (EV) control. Similarly, in the knockdown experiments, the negative control (NC) group exhibited significantly higher viability compared to the XBP1-knockdown (sh) group (*n* = 3). **G** Fold Viability of A549 cells after treatment with various concentration of ES. The OE group was able to maintain cell viability at higher ES concentrations, whereas the sh group exhibited increased sensitivity to ES in a dose-dependent manner. **H** Bar plot showing EdU positive ratio of A549 cells in four groups (OE, EV, sh, and NC) after treatment with 40 nM and 100 nM ES for 72 h (*n* = 3). **I** Representative fluorescence images of EdU staining. The OE group maintained significantly higher proliferation levels under ES treatment. **J** Representative images of tumor tissues from xenograft mice with OE or EV group under ES treatment or control conditions. Adjacent graph shows tumor growth curves and volume bar chart, highlighting the effect of XBP1s overexpression on tumor progression and cuproptosis resistance. (*n* = 6 per group). **H** High-resolution Transmission Electron Microscopy (TEM) images (×8.0k and ×25.0 k magnifications) were acquired using a Hitachi HT7700 microscope. The EV group exhibited a higher number of mitochondria compared to the OE group, but with abnormal morphology, such as loss of mitochondrial cristae and swelling. Despite these observations, no significant differences were detected in the statistical analysis (*n* = 3). The data are presented as the mean ± SEM. *p < 0.05, **p < 0.01, ***p < 0.001, ****p < 0.0001.

We then utilized elesclomol (ES), a copper-loaded ionophore, to simulate a high-copper environment and induce cuproptosis in LUAD cells. Western blot analysis showed a significant increase in the spliced form-XBP1s but not XBP1u, after ES treatment (Fig. 3A). This suggests that XBP1s may play a role in the resistance of LUAD cells to cuproptosis. Higher expression of XBP1s was confirmed to be associated with poorer pathological subtypes by tissue microarray (Fig. S1G, H, Fig. 3B, and Fig. 3E). These results suggest that tissues of higher pathological grade LUAD may have higher copper concentrations and a significant increase in XBP1s. Additionally, we performed a paired analysis of XBP1s expression between normal and tumor tissues (Fig. 3C, D).

To determine whether the overexpression of XBP1s was related to cell death caused by higher copper content, the viability of A549 and PC9 cells was assessed 72 h after ES (40 nM and 100 nM)-CuCl_2_ pulse treatment. The results showed that the overexpression of XBP1s rescued cells from cuproptosis (Fig. 3F and Fig. S2A). We then performed a cell viability assay based on the concentration of ES (Fig. 3G and Fig. S2B). In addition, XBP1s overexpression significantly reduced the inhibitory effect of ES on cell proliferation, as shown by the the 5-ethynyl-2′-deoxyuridine (EdU) incorporation assay (Fig. 3H, I and Fig. S2C-D). Moreover, xenograft animal models confirmed that the overexpression of XBP1s significantly inhibited cuproptosis caused by ES in vivo (Fig. 3J, K). We also utilized transmission electron microscopy (TEM) to investigate the alterations in mitochondrial morphology in A549 cells following ES treatment. Interestingly, the XBP1s overexpression group exhibited a reduced number of mitochondria, but with relatively normal morphology. In contrast, the EV group had a higher number of mitochondria, which were disorganized, swollen, and exhibited loss of mitochondrial cristae (Fig. 3L).

### XBP1s undergoes phase separation in vitro and in vivo

**Fig. 4:**
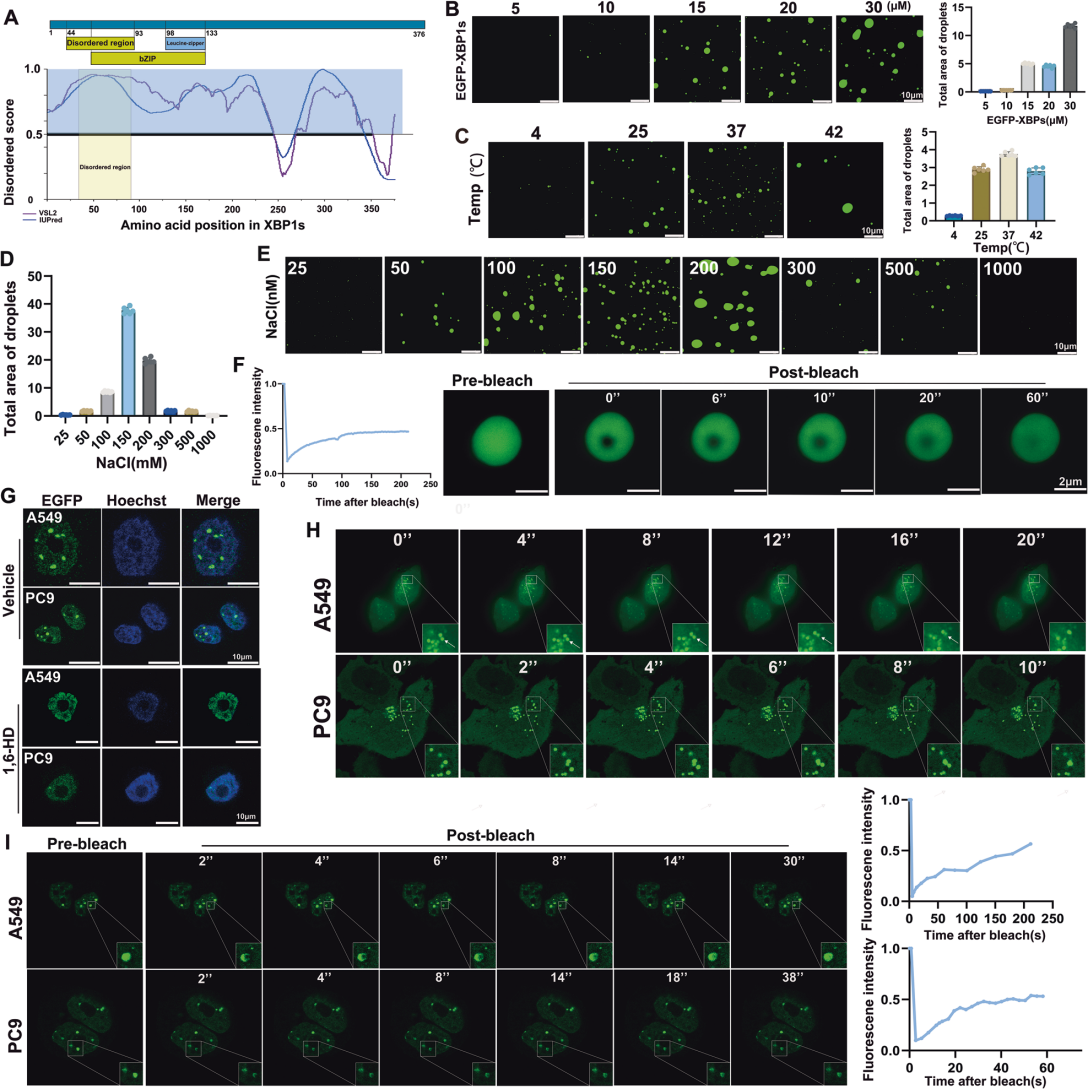
XBP1s undergoes phase separation in vitro and form condensates in the nucleus. **A** Domain structure and graphs of XBP1s IDRs based on VSL2 and IUPred algorithms. Scores >0.5 indicate disorder. Yellow shade depicts the designated core IDR (cIDR). **B** Representative fluorescence images (488 nm) of XBP1s droplets at varying protein concentrations (5 µM–30 µM) in a buffer containing 150 nM NaCl and 20% PEG-8000 (default condition unless otherwise specified). Both the size and number of droplets increased as protein concentration rose. Quantification of droplet area is shown on the right. **C** Representative fluorescence images (488 nm) of XBP1s-droplets at different temperatures. The droplet number peaked at 37 °C. Quantification of droplet area is shown on the right. **D**, **E** Representative fluorescence images (488 nm) of XBP1s-droplets at different NaCl concentrations (25–1000 nm). At 150 mM NaCl, droplet numbers were the highest, while at 200 mM NaCl, droplets were larger but fewer, indicating a concentration-dependent phase separation behavior. **F** Fluorescence recovery after photobleaching (FRAP) analysis of EGFP-XBP1s droplets in vitro. The graph illustrates the recovery of fluorescence intensity within 0–60 s, indicating the liquid-like dynamics of the droplets. **G** Live-cell imaging of A549 and PC9 cells, EGFP-XBP1s puncta were observed under treatment with buffers, both in the presence and absence of 5% 1,6-hexanediol. Nuclei were distinctly visualized through Hoechst staining. **H** Live-cell imaging showed the fusion phenomenon of XBP1s droplets was observed through live-cell imaging in A549 and PC9 cells, indicating the liquid-like dynamics of the droplets. **I** FRAP recovery of EGFP-XBP1s puncta in live A549 and PC9 cells. White squares mark photo-bleached puncta. The fluorescence recovery occurred within 2–30 s in A549 cells and 2–38 s in PC9 cells, further confirming the dynamic and liquid-like properties of XBP1s condensates. Quantification of fluorescence recovery is displayed on the right.

The full-length XBP1s protein contained an intrinsically disordered region (IDR) that is important for interactions with other proteins (Fig. 4A). Proteins with a wide range of IDR tend to undergo LLPS, a biochemical phenomenon that plays an important role in cellular physiology [22]. We investigated whether XBP1s exhibits this property. The droplet assay showed that purified EGFP-XBP1s can spontaneously form droplets in solution. These droplets became larger and more intense under specific physicochemical conditions, such as protein concentration, temperature and salt concentration (Fig. 4B–E). Moreover, droplet formation was significantly inhibited by 5% 1,6-hexanediol (1,6-HD), a compound known to disturb LLPS by disrupting weak hydrophobic interactions (Fig. S3A). We also observed fusion events between two adjacent droplets and fluorescence recovery of the droplets within 120 s after the photobleaching treatment (Fig. S3B and Fig. 4F). These results show that XBP1s is capable of forming condensates exhibiting liquid-like characteristics in vitro.

To test whether XBP1s can undergo intracellular LLPS, we ectopically expressed EGFP-XBP1s in A549 and PC9 cells. As expected, XBP1s formed condensates in the nucleus that were disrupted by 1,6-HD (Fig. 4G). Similar to the in vitro experiments, we observed fusion events of droplets formed by XBP1s (Fig. 4H). Fluorescence recovery after photobleaching (FRAP) also confirmed the liquid-like properties of XBP1s puncta (Fig. 4I). We wondered whether this characteristic of LLPS in XBP1s was enhanced when LUAD cells were under copper-induced stress. After ES was applied to LUAD cell lines, live cell imaging was performed using confocal microscopy (×63). The results showed that ES treatment significantly increased the number and dimensions of the droplets (Fig. S4A-B).

We purified mutant GFP–XBP1s with deletion of the IDR and XBP1s-IDR to identify role of the IDR in XBP1s and analyzed by a droplet assay. XBP1s-IDR alone could undergo LLPS in vitro, whereas the removal of the IDR of XBP1s considerably reduced droplet formation (Fig. S3C–D). Furthermore, we ectopically expressed EGFP-XBP1s-IDR in LUAD cell lines and observed the formation of nuclear droplets by XBP1s-IDR which were greatly suppressed after 1,6-HD treatment (Fig. S3F–G). To verify the liquid-like properties, we selected typical droplets in vitro for FRAP experiments in vitro and in cells (Fig. S3E and H). Although the purified IDR was able to undergo fluorescence recovery, the time was significantly longer compared to the full-length XBP1s protein.

### XBP1s compartmentalizes SEs-related coactivators in the nucleus

**Fig. 5:**
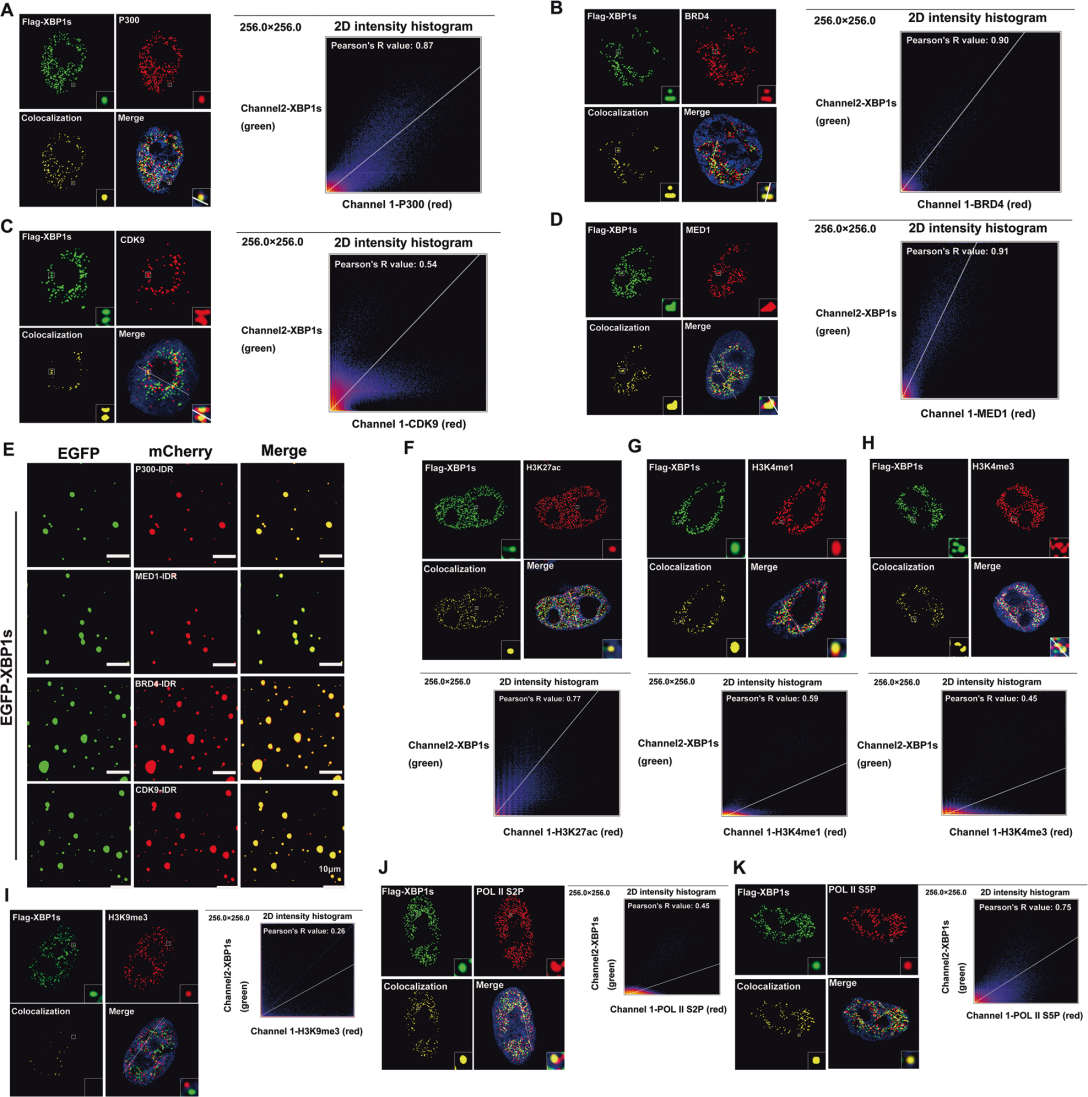
XBP1s compartmentalizes SEs-related coactivators to nuclear puncta. Colocalization of transcriptional coactivators P300 (**A**), BRD4 (**B**), CDK9 (**C**), MED1 (**D**) with Flag-XBP1s (green) in nuclear puncta in A549 cells. Line scans of the colocalization images are depicted by white arrows with quantification (2D intensity histograms) shown to the right, highlighting Pearson’s correlation coefficients (R-values). Areas of colocalization are indicated in yellow. **E** Representative images of droplet formation of EGFP-XBP1s (488 nm) with mCherry-P300-IDR, mCherry-MED1-IDR, mCherry-BRD4-IDR or mCherry -CDK9-IDR (594 nm). The co-localization of EGFP-XBP1s (green) and mCherry-labeled IDR proteins (red) demonstrates their phase separation compatibility and mutual incorporation into condensates. Localization of active histone markers H3K27ac (**F**), H3K4me1 (**G**) and H3K4me3 (**H**), repressive histone markers H3K9me3 (**I**) and active RNA POI II S2P (**J**) and S5P (**K**) (red)with Flag-XBP1s (green) in A549 cells, which suggested that the condensates formed by XBP1s are associated with the transcriptional activation of target genes. Line scans of the colocalization images are depicted by white arrows with quantification (2D intensity histograms) shown to the right and below, highlighting Pearson’s correlation coefficients (R-values). Areas of colocalization are indicated in yellow.

LLPS is believed to be the basis for the formation of membrane-less organelles (MLOs) in eukaryotic cells. Since the first MLO was observed within the nucleus of neuronal cells, many such MLOs have been discovered in the nucleus, cytoplasm, and on membranes of essentially all eukaryotic cells. Examples include nucleoli, Cajal bodies, and PML bodies in the nucleus as well as stress granules and P bodies in the cytoplasm [23–25]. We examined the specific components and functions of nuclear puncta formed by XBP1s. Colocalization studies indicated that the XBP1s condensates did not contain markers of PML bodies, nucleoli, or Cajal bodies (Fig. S4C–E). XBP1s has previously been shown to recruit the co-activator P300 to activate gene transcription [26]. Therefore, we speculated that LLPS of XBP1s may tend to form SEs that can activate the transcription burst of target genes. Oncogenic SEs are distinguished from regular enhancers by their higher densities of coactivator and mediator binding, as well as gene activation histone markers [12]. Consistent with our hypothesis, immunostaining analyses showed that XBP1s colocalized with P300, MED1, BRD4, and CDK9, which were biomarkers of SEs (Fig. 5A–D). Furthermore, EGFP-XBP1s could form droplets with mCherry-fused P300, MED1, and BRD4 in vitro (Fig. 5E). To confirm the link between XBP1s and transcription, we performed immunofluorescence analysis of XBP1s and histone modification markers. As expected, Flag-XBP1s showed puncta that overlapped with the gene activation histone markers H3K27ac, H3K4me1, and H3K4me3, but not with the repressive marker H3K9me3 (Fig. 5F–I). Ser2 and Ser5 phosphorylation of the C-terminal heptapeptide repeats of RNA polymerase II (Pol II S2P and S5P, respectively) are general markers of gene transcription. Flag-XBP1s colocalized with Pol II S2P and S5P signals, suggesting XBP1s-related SEs also contain active RNA polymerase II (Fig. 5J, K). We also conducted the colocalization detection of XBP1s and SE-related transcriptional elements in the PC9 cell line (Fig. S5A–J). The DAPI staining images are shown in Fig. S5K–L.

### XBP1s promotes the formation of SEs clusters to drive MGRN1 transcription under copper stress environment

To elucidate the relationship between XBP1s-associated SEs and target gene expression, we employed Cleavage Under Targets and Tagmentation (CUT&Tag)-seq on SEs markers (P300, BRD4, MED1, H3K27ac) across varying copper stress conditions (vector, 40 nm and 100 nm ES), to identify the precise localization of XBP1s binding sites on genomic chromosomes. At higher copper concentrations, XBP1s was enriched in several genes related to ubiquitin-mediated proteolysis (Fig. S6). Recognizing the link between copper-mediated cell death and the accumulation of abnormally acylated proteins, we surmised that XBP1s might activate ubiquitination-related genes under conditions of high copper stress, thereby facilitating the degradation of abnormally accumulated proteins. In particular, MGRN1, which encodes an E3 ubiquitin-protein ligase increased in parallel with the overexpression of XBP1. In addition, the protein and mRNA levels of MGRN1 were simultaneously elevated when we overexpressed XBP1s (Fig. S8A). Two XBP1s binding sites (S1 and S2) were found in the transcription start site (TSS) of MGRN1 gene (Fig. S7A). We performed CUT&Tag-qPCR to evaluate the effects of XBP1s on the binding of co-activators to the MGRN1 promoter. The results showed that coactivators bind to both S1 and S2, but S2 appears more prominent (Fig. S7B–C). Three reporter constructs were generated using Gaussia luciferase (Gluc) driven by WT-TSS, S1M, and S2M. Mutagenesis of S1 and S2, especially the latter, caused a remarkable reduction in MGRN1 promoter activity in reporter assays (Fig. S7D). We speculated whether the copper environment affected the interaction between the transcription elements and TSS of MGRN1. CUT&Tag-qPCR was performed with different concentrations of ES treatment for transcription elements and gene activation histone markers H3K27ac, H3K4me1, and H3K4me3. Interestingly, elemental binding on S1 was roughly unchanged, but binding on S2, especially XBP1s and BRD4, was significantly elevated after ES treatment (Fig. S7E–F). We also observed a significant increase in histone acetylation at both the S1 and S2 positions after ES treatment, suggesting that copper ac mulation may activate the transcription of MGRN1 (Fig. S7G and Fig. S8B–C).

**Fig. 6:**
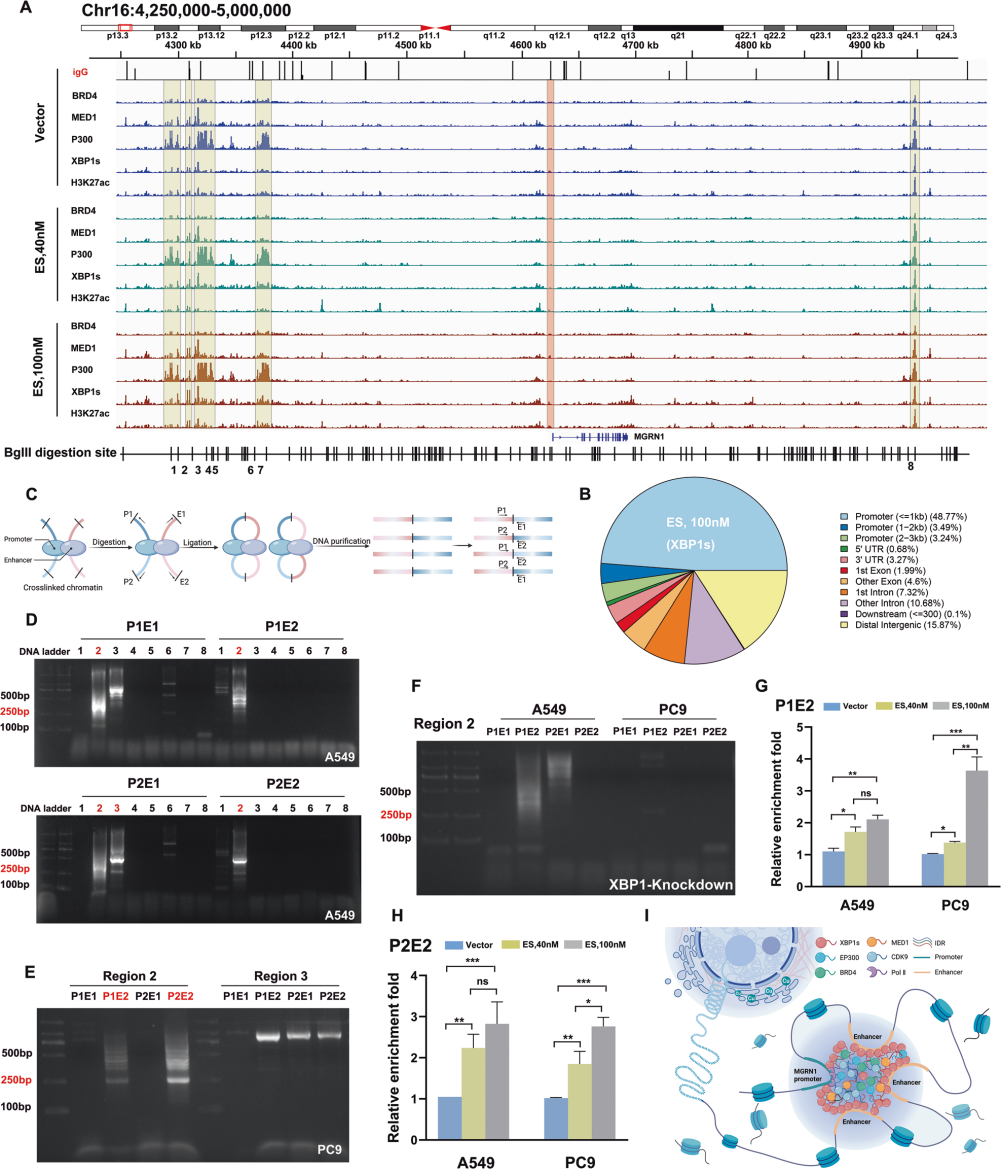
XBP1s condensates enhance the interaction between the MGRN1 promoter and distal enhancer especially under copper stress. **A** Schematic view across the MGRN1 gene locus (chr16:4, 250,000–5,000,000) with genomic and epigenetic information. Graphic active regulatory regions were generated from CUT&Tag data of XBP1s, P300, MED1, BRD4 and H3K27ac, respectively treatment with ES at different concentrations (Vector, 40 nM and 100 nM). Delineate potential super-enhancer sites through the identification of shared enrichment domains across overlapping image regions. The BgIII digestion sites and corresponding fragments are numbered below the graph. **B** CUT&Tag sequencing results depicting the genomic elements enriched with XBP1s under ES 100 nM treatment. The pie chart illustrates the distribution of XBP1s-binding regions across various genomic features, including promoters, enhancers, 5’ U TRs, 3’ UTRs, exons, and intergenic regions. **C** Workflow diagram of Chromatin conformation capture (3 C), illustrating DNA crosslinking, digestion, ligation, and DNA purification steps. P1 and P2 represent the two ends of the MGRN1 promoter, while E1 and E2 represent the two ends of the potential enhancer regions. Four possible primer combinations (P1E1, P1E2, P2E1, P2E2) were designed to assess physical interactions between the promoter and enhancer regions. If a physical interaction exists, PCR amplification of the 3 C products will yield DNA fragments of the expected size (about 250–350 bp), which can be visualized as distinct bands using agarose gel electrophoresis. **D** Agarose gel electrophoresis of 3 C products from A549 cells, targeting 8 potential regions identified based on (**A**) and BglII digestion sites. Each region was tested for interaction with the MGRN1 promoter using primer combinations P1E1, P1E2, P2E1, and P2E2. The results indicate that region 2 produces bands of the expected size for all primer combinations (P1E1, P1E2, P2E1, and P2E2), while region 3 only produces a band for P2E1. Other regions were excluded due to the absence of bands of the correct size. **E** 3 C experiments repeated in PC9 cells for regions 2 and 3. Results reveal that only region 2 exhibits correctly sized positive bands for P1E2 and P2E2, while no such bands are observed for region 3. **F** Knockdown of XBP1 significantly reduced the interaction between region 2 and the MGRN1 promoter, as demonstrated by diminished PCR band intensity in both A549 and PC9 cells. **G**, **H** qPCR analysis of the 3 C products under various treatment conditions (Vector, 40 nM and 100 nM) revealed that copper stress significantly enhanced the interaction intensity between the MGRN1 promoter and region 2 in both A549 and PC9 cells. Statistical analysis was performed using one-way ANOVA followed by Tukey’s post-hoc test. *P < 0.05, **P < 0.01, ***P < 0.001, ****P < 0.0001, ns not significant. Error bars represent mean ± SD from three independent experiments. **I** A refined depiction of an enhancer cluster architecture, facilitated by XBP1s-mediated phase separation, encompasses the integration of coactivators and is reinforced by distal enhancers, collectively orchestrating the potentiation of MGRN1 gene transcription.

Simultaneously, we identified several possible SE regions enriched in XBP1s and other SEs markers (Fig. 6A). The results, shown in the pie chart (Fig. 6B and Fig. S8D), indicate the relative distribution of peaks across different genomic regions, including exons, introns, 5’ UTR, 3’ UTR, and intergenic regions. The chromosome conformation capture (3 C) approach [27] with restriction enzyme digestion, digested genomic DNA, DNA ligation, and PCR amplification of ligated DNA was used to examine the neighboring regions in the formation of enhancer complexes by interacting with the MGRN1 promoter (Fig. 6C). After BgIII digestion, eight optimal cleavage sites were selected to investigate their interactions with the promoters. Most ligation-dependent PCR amplifications were manifested by the BgIII-digested segments around region 2 and 3 in A549 cells (Fig. 6D).

However, we observed inconsistency of PCR amplified regions between A549 and PC9 samples, which helped us excluding the region 3 due to the bands not matching the expected size and we confirmed the P1E2 and P2E2 of region 2 (Fig. 6E). Furthermore, shRNA-mediated XBP1 knockdown eliminated most of the PCR bands, indicating that the physical proximity between the MGRN1 promoter and enhancer is dependent on the presence of XBP1s (Fig. 6F). To investigate the effects of different copper concentrations on the interaction between the MGRN1 promoter and enhancer, we performed quantitative PCR analysis. Our findings reveal that ES treatment significantly enhanced the physical interaction between them, particularly for P2E2 of region 2 (Fig. 6G, H). Figure 6I illustrates the mechanism by which XBP1s-dependent SEs regulate MGRN1 transcription.

### E3 ubiquitin-protein ligase MGRN1 promotes K48-linked ubiquitination of LIPT1

**Fig. 7:**
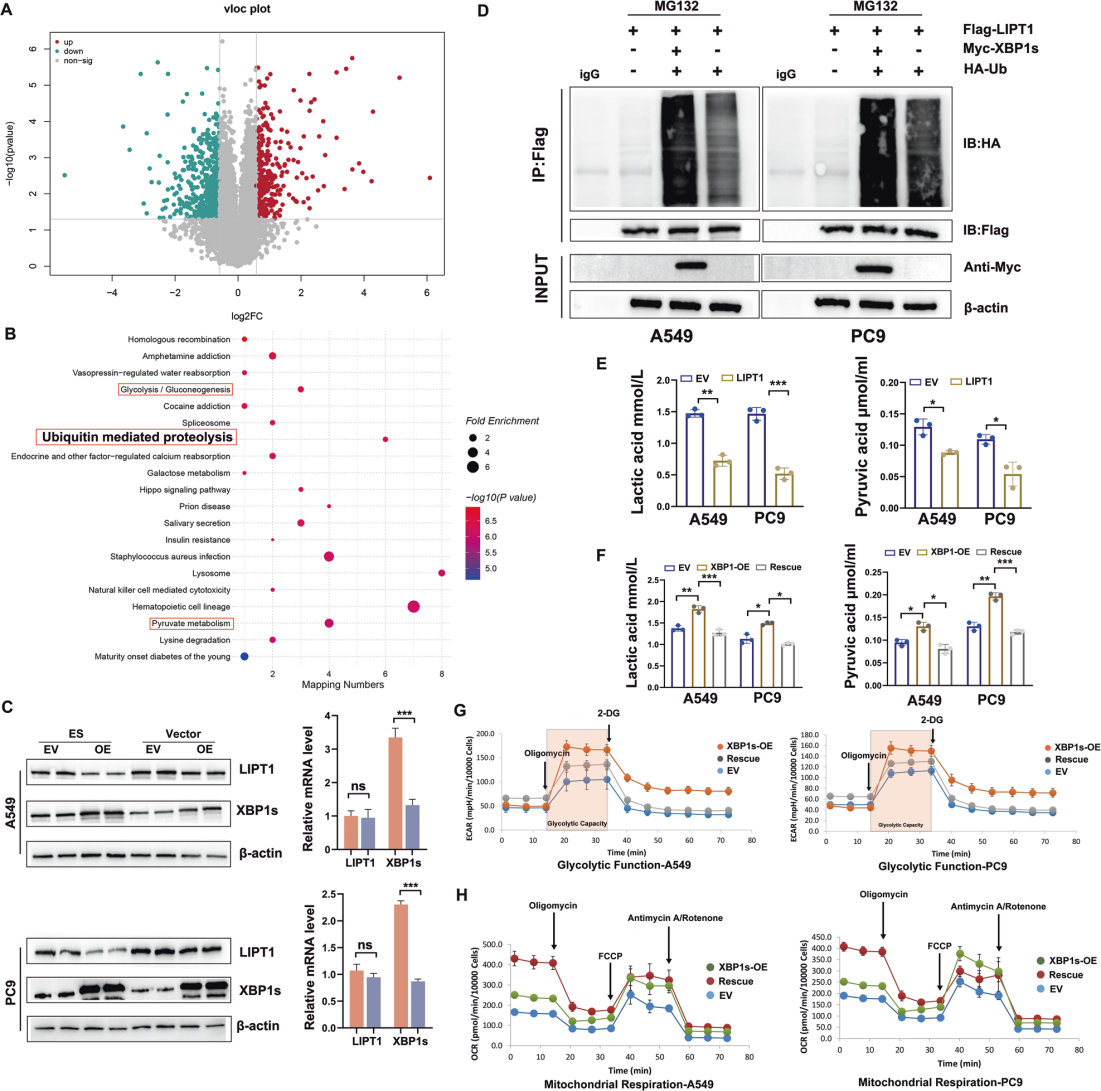
XBP1s promotes LIPT1 ubiquitination and alters glycolysis and mitochondrial respiration. **A** Volcano plot showing differential protein expression profiles in A549 cells overexpressing XBP1s (XBP1s-OE) compared to the expression vector (EV) control after treatment with 40 nM ES. Red and green dots represent significantly upregulated and downregulated proteins, respectively (log2FC > 1.5, -log10 *p*-value > 1.3). Notably, LIPT1 is significantly downregulated. **B** KEGG pathway enrichment analysis of significantly altered proteins, highlighting activated glycolysis, ubiquitin-mediated proteolysis, and pyruvate metabolism pathways. **C** Western blot (WB) and quantitative PCR (qPCR) analyses of LIPT1 and XBP1s expression in LUAD cells with XBP1s overexpression (OE) compared to EV. LIPT1 protein levels are reduced in XBP1s-OE cells, while mRNA levels remain unchanged. β-actin was used as a loading control. **D** Immunoprecipitation (IP) assay demonstrating enhanced ubiquitination of LIPT1 in the presence of XBP1s in LUAD cells. Cells were treated with MG132 to inhibit proteasomal degradation. Flag-LIPT1, Myc-XBP1s, and HA-Ub were co-expressed, and ubiquitinated LIPT1 was detected using anti-HA antibodies. **E** Lactic acid and pyruvic acid levels in LUAD cells overexpressing LIPT1. Overexpression of LIPT1 reduces both metabolites (*n* = 3). **F** Lactic acid and pyruvic acid levels in LUAD cells with XBP1s overexpression (OE) or rescue of LIPT1 expression (Rescue). XBP1s-OE increases glycolysis metabolites, while LIPT1 rescue reduces their levels (*n* = 3). Data are shown as mean ± SD, *P < 0.05, **P < 0.01, ***P < 0.001, ***P < 0.0001, ns not significant. **G** Extracellular acidification rate (ECAR) measurements in LUAD cells. XBP1s-OE enhances glycolysis, while LIPT1 rescue reverses the effect. Shaded areas indicate glycolytic function after oligomycin addition. Data are presented as mean ± SD. **H** Oxygen consumption rate (OCR) analysis of mitochondrial respiration in LUAD cells. XBP1s-OE suppresses mitochondrial respiration, while LIPT1 rescue restores respiration levels. OCR values after sequential addition of oligomycin, FCCP, and antimycin A/rotenone are shown. Data are presented as mean ± SD.

Drawing upon existing research that identified seven key regulators (FDX1, LIPT1, LIAS, DLD, DLAT, PDHA1, and PDHB) of cuproptosis through genome-wide CRISPR-Cas9 screening [5], and our CUT&Tag findings which indicated enrichment of the ubiquitin-proteasome pathway under copper stress conditions, we hypothesized that XBP1s may facilitate the degradation of critical proteins that support cuproptosis. We performed 4D-DIA (Data independent acquisition) proteomics analysis to explore the alterations in intracellular protein expression patterns elicited by XBP1s overexpression, specifically under copper stress (Fig. S9A–E). This approach aimed to reveal how XBP1s modulate the cellular proteome in response to copper stress, providing valuable insights into its regulatory role. The analysis of proteomics showed that the LIPT1 content was significantly lower in the XBP1s-OE group than in the control group (Fig. 7A). Using western blotting and RT-qPCR, we validated that XBP1s negatively regulates the protein level of LIPT1, but not at the RNA level. Interestingly, LIPT1 levels were significantly reduced after ES treatment (Fig. 7C), suggesting that XBP1s exerts a stronger effect on LIPT1 in the context of Cu accumulation. Additionally, GSEA showed that XBP1s-group is also enriched in the ubiquitin-mediated proteolysis pathway (Fig. 7B). Immunoprecipitation (IP) and immunoblotting (IB) analyses revealed a significant increase in LIPT1 ubiquitination levels when co-transfected with XBP1s (Fig. 7D).

Furthermore, we repeated the cell viability and EdU incorporation assays on the XBP1s and LIPT1 dual-expressing LUAD cell lines. LITP1 overexpression abolished the protective effects of XBP1s in two LUAD cell lines under copper stress (Fig. S2E–H and Fig. S11A–D). After overexpressing LIPT1 and treating A549 cells with ES, the mitochondrial morphology exhibited increased numbers, swelling, disorganization, and a loss of cristae, in contrast to the XBP1s-OE group (Fig. S11E). Briefly, we found that XBP1s may rescue LUAD cells from cuproptosis by promoting the ubiquitination and degradation of the cuproptosis-related gene LIPT1.

Based on the above results, we speculated that XBP1s may promote the ubiquitination of LIPT1 via MGRN1. As shown in Fig. S10A and S10B, MGRN1 interacts with endogenous and exogenous Flag-LIPT1. Co-expression of His-MGRN1 and HA-ubiquitin with Flag-LIPT1 revealed that LIPT1 was extensively ubiquitinated upon co-expression with MGRN1 (Fig. S10C). Consistently, the ubiquitination of LIPT1 was almost abolished when we downregulated MGRN1 by shRNA (Fig. S10D). Lys48-linked ubiquitin chains serve as the main target signals for protein degradation by the proteasome, whereas Lys63-linked ubiquitin chains are involved in multiple cellular events that do not rely on proteasome-mediated degradation pathways [28]. We speculated which type of polyubiquitin modification on the LIPT1 protein was affected by MGRN1 and coexpressed lys48-resistant (Lys48R) and lys63-resistant (Lys63R) forms of ubiquitin with His-MGRN1. The results showed that co-transfection with lys48R significantly reduced the ubiquitination LIPT1 whereas co-transfection with lys63R had no effect (Fig. S10E–F). In summary, lys48-linked polyubiquitination mediated by MGRN1 is critical for the degradation of LIPT1.

### Glycolysis metabolism was enhanced during LIPT1 deficiency

Loss of LIPT1 inhibits the TCA cycle, forcing tumor cells to augment glycolysis to acquire energy essential for growth [29]. Notably, cells with a heightened dependence on mitochondrial respiration demonstrated profound sensitivity to copper ionophores, approaching a 1000-fold increase compared to cells reliant on glycolysis [5]. This compelling finding prompted the speculation that XBP1s-mediated downregulation of LIPT1 may elicit metabolic reprogramming in LUAD cells, shifting towards augmented glycolysis and suppressed aerobic respiration, thereby mitigating copper-mediated cell death. As expected, overexpression of LIPT1 reduced the extracellular levels of L-lactate and pyruvate in the cell culture media after ES treatment, whereas overexpression of XBP1s produced the opposite outcome (Fig. 7E, F). The glycolytic stress test indicated that up-regulation of XBP1s increased extracellular acidification rate (ECAR) levels (Fig. 7G). Oxygen consumption rate (OCR) analysis showed that LIPT1 overexpression enhanced cellular oxygen consumption (Fig. 7H).

Previous study confirmed that DLAT, another pivotal regulator of cuproptosis, can bolster cellular glycolysis [30]. Intrigued by this finding, we conducted western blot analysis and were surprised to discover significantly elevated levels of DLAT protein in the XBP1s overexpression group (Fig. S9F). This finding underscores the critical influence of XBP1s on the metabolic reprogramming of tumor cells, although the precise mechanism underlying XBP1s-mediated upregulation of DLAT remains elusive.

### XBP1s downregulated LIPT1 via MGRN1 to inhibit cuproptosis in vivo

To further investigate the axis of XBP1s-MGRN1-LIPT1 in resisting cuproptosis, we subcutaneously injected 1 × 10^7^ XBP1s-OE A549 cells (suspended in a final volume of 100 µL) into each mouse. The vehicle used for the injections was phosphate-buffered saline (PBS). Mice were divided into ES treatment groups: control, MGRN1-knockdown (MGRN1-KD), control, control+JQ1 (an SEs inhibitor) and untreated groups: control, MGRN1-KD. A total of *n* = 6 mice per group (3 male and 3 female) were used for each experiment. The 50 mg/kg dose of ES was administered via peritumoral injection every three days.

**Fig. 8:**
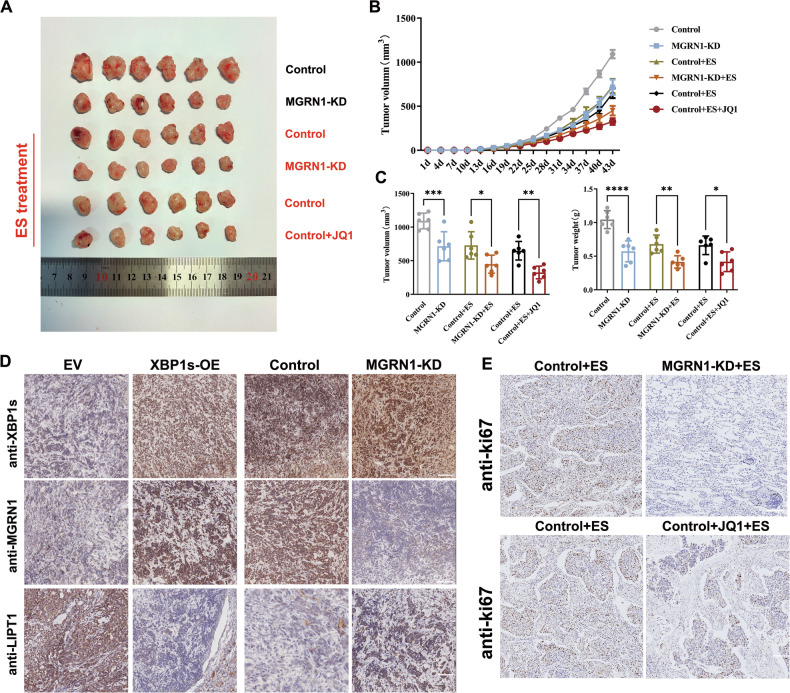
Combination with SE inhibitors significantly enhanced the tumor-suppressing effect of ES in *xenograft* model. **A** Representative images of tumor samples from xenograft mice under different treatment conditions (Control, MGRN1-KD, ES+Control, ES + MGRN1-KD, ES+Control and ES + JQ1). **B**, **C** Quantitative analysis of tumor volume and weight. Tumor volume was measured every three days, and tumor weight was recorded at the endpoint. Data are presented as mean ± SD, with significant differences denoted by *P < 0.05, **P < 0.01, ***P < 0.001, ****P < 0.0001. **D** Immunohistochemical staining of XBP1s, MGRN1, and LIPT1 in tumor tissues, using previously described tumors (from Fig. 3J) and newly generated tumors in this experiment. These results validate the regulatory relationship among XBP1s, MGRN1, and LIPT1. **E** Ki67 staining of *xenograft* tumor tissues to evaluate the impact of XBP1s-related SEs on cell proliferation.

Tumor growth was monitored, and results showed that mice implanted with XBP1s-OE A549 cells developed significantly larger tumors, whereas MGRN1-knockdown cells failed to maintain their resistance to copper-induced cell death. In order to explore whether inhibiting SEs of XBP1s potentiates the sensitivity of tumor cells to cuproptosis in vivo, we treated mice with either vehicle or JQ1. As anticipated, dual treatment with ES and JQ1 significantly slowed tumor growth and reduced tumor volume compared to the single treatments, highlighting the potential of combination therapies (Fig. 8A–C).

Immunohistochemical analysis revealed a correlation between the expression of XBP1s, MGRN1, and LIPT1 in the mouse tumors (Fig. 8D), which opens new possibilities for the development of targeted therapies aimed at exploiting cuproptosis pathways in LUAD. To further evaluate tumor proliferation, we performed Ki-67 staining on tumor sections. As shown in Fig. 8E, Ki-67 expression was significantly reduced in the tumors treated with ES, particularly in the MGRN1-KD group and the JQ1 combination treatment group. These results suggest that the reduced tumor growth observed in these groups is associated with a decrease in tumor cell proliferation, correlating with the tumor volume differences.

## Discussion

Copper homeostasis plays an important role in tumor growth, invasiveness, and resistance to chemotherapy [31, 32]. Excessive intracellular copper accumulation triggers a unique form of cell death called cuproptosis, facilitated by an evolutionarily conserved pathway involving protein lipoylation. Cells with active TCA cycles exhibit elevated lipoylated enzymes, such as the pyruvate dehydrogenase complex, where the lipoyl moiety directly chelates copper, causing protein aggregation and acute proteotoxic stress, ultimately leading to cell death [5]. Genetic variation in copper homeostasis leads to life-endangering disorders. Specifically, copper ionophores have been proposed as promising anticancer agents [33–36], so do the copper chelators [37–40]. These compounds exhibit potential in combating cancer cells by modulating the balance of copper within cells, offering a potential avenue for cancer treatment research.

Our study revealed that LUAD tumor tissues exhibit significantly higher copper levels than normal tissues, with copper content positively correlated to pathological grade, particularly grade 3 tumors. This finding highlights the potential of targeting copper accumulation in LUAD treatment strategies. However, contrary to common expectations, our results show that cuproptosis is not significantly induced in advanced LUAD, suggesting a novel regulatory mechanism. Single-cell RNA sequencing analysis helped us finding UPR activation in high-grade tumors. Interestingly, it has been proven in research that another effector molecule of the UPR, ATF4, is related to the inhibition of cuproptosis [21]. The UPR pathway is initiated by ER stress sensors that detect accumulation of unfolded or misfolded proteins in the ER [14]. XBP1s has been found to be associated with proteostasis reprogramming, mitochondrial quality control, and lipid metabolism in a variety of diseases [41–43]. In tumors, XBP1s has been shown to be related to tumorigenesis, activation of abnormal oncogenic pathways, and tumor drug resistance [44–46]. Additionally, in LUAD, XBP1s has been identified as a potential target to mitigate chemotherapy resistance [47]. We found that XBP1s significantly inhibited cuproptosis of LUAD cells in vivo and in vitro. To sum up, our findings highlight the dual role of copper and the UPR pathway, particularly XBP1s, in LUAD progression. As demonstrated in Fig. 1, we observed tumor samples with high copper concentrations but low proliferation rates, underscoring the heterogeneous nature of LUAD. Moreover, single-cell analysis revealed that within epithelial cells, the expression of cuproptosis-related gene sets in tumor cluster Epi-C2 was higher in low-grade LUAD compared to high-grade cases. Although these observations may be influenced by sample numbers or other factors, they reflect the inherent heterogeneity of LUAD and the complexity of its response to copper. This heterogeneity poses a significant challenge for identifying the tumor cell populations most relevant to copper metabolism and targeting them therapeutically. Future studies leveraging larger sample sizes and advanced spatial transcriptomics are needed to pinpoint the specific tumor subpopulations sensitive to copper dysregulation, paving the way for more precise therapeutic strategies.

LLPS in various subcellular regions facilitates the formation of membraneless condensates, compartmentalizing biomolecules like proteins, RNAs, and DNAs to enable distinct biological activities [25]. The XBP1s protein contains a zinc finger region and an IDR, which promotes LLPS. Our experiments demonstrated its ability to undergo LLPS both in vitro and in cells, with its IDR forming condensates independently. Notably, copper stress enhanced the size and number of XBP1s droplets, suggesting a direct regulatory effect of copper on XBP1s condensates.

The seminal discovery by Young’s group involved the application of phase separation in transcriptional regulation [9, 48]. They demonstrated the formation of phase-separated condensates that enclosed multiple coactivators, effectively functioning as SEs, which represents a significant advancement in our understanding of transcriptional processes. As a transcription factor, XBP1s has been reported to recruit the coactivator P300 to activate target gene transcription [26]. After excluding other potential condensates within the nucleus, we assumed that the droplets formed by XBP1s were SEs. Immunofluorescence staining confirmed the spatial colocalization of XBP1 with SE-related coactivators and mediators, indicating their close proximity within the cell. CUT&Tag-seq analyses under varying copper conditions revealed temporal specificity in SEs formation, with high copper levels enriching the ubiquitin-proteasome pathway, potentially linked to the degradation of misfolded proteins. WB and PCR analyses demonstrated a positive correlation between MGRN1 and XBP1s at both the protein and mRNA levels. MGRN1, an E3 ubiquitin ligase, mediates proteasomal degradation of target proteins. XBP1s, P300, MED1 and BRD4 shared two overlapping binding sites (S1 and S2) within the MGRN1 promoter region. While copper minimally affected S1 binding, it significantly enhanced transcriptional element affinity for S2. Our data identified SEs spanning a 750 kb region near MGRN1, characterized by dense enhancer clusters and H3K27ac enrichment. Copper accumulation intensified promoter-enhancer interactions mediated by LLPS condensates, underscoring the dynamic regulation of SEs by copper-modulated XBP1s activity.

4D-DIA proteomics revealed that XBP1s significantly reduced LIPT1 protein levels without altering its mRNA expression, suggesting a post-translational modification mechanism. Immunoprecipitation confirmed that MGRN1 interacts with LIPT1 and enhances its K48-linked ubiquitination. Interestingly, single-cell sequencing analysis revealed enhanced glycolysis in high-grade LUAD, whereas proteomics indicated that XBP1s promoted glycolysis. These findings suggest that XBP1s inhibits cuproptosis by inducing metabolic reprogramming. Loss of LIPT1 disrupts the TCA cycle [29], potentially forcing tumor cells to rely on glycolysis, while making them less sensitive to copper-dependent cell death [5]. Experimental results further support this hypothesis: overexpression of LIPT1 reduced glycolysis markers and increased oxygen consumption, while XBP1s overexpression had the opposite effect. Moreover, XBP1s upregulated DLAT protein levels, another key glycolysis regulator, further emphasizing its role in metabolic reprogramming. However, the mechanism by which XBP1s regulates DLAT remains unclear.

The SEs inhibitor JQ1 has demonstrated the ability to enhance the efficacy of clinically established first-line therapies [49]. ES, a copper ionophore, initially showed promise in clinical trials for melanoma but was later terminated due to insufficient efficacy in subsequent studies [36]. However, recent insights into the role of copper in cancer biology, particularly its involvement in cuproptosis, have reignited interest in ES as a therapeutic agent. In our study, the combination of ES and JQ1 drastically impaired the capacity of XBP1s to counteract cuproptosis, suggesting a synergistic effect in targeting LUAD metabolic and transcriptional vulnerabilities. This combination approach could potentially overcome the limitations of ES observed in earlier trials, particularly in tumors characterized by aberrant XBP1s activation and metabolic reprogramming. Future studies may focus on identifying biomarkers, such as copper content or XBP1s activity, to stratify patients who are most likely to benefit from the combination. Additionally, preclinical and clinical investigations are needed to evaluate the safety, efficacy, and potential combinatory effects of JQ1 and ES in LUAD, ultimately paving the way for personalized therapies targeting the metabolic vulnerabilities of this cancer type.

Despite the significant advancements presented in this study, several limitations should be acknowledged. First, our use of a single copper ionophore limits the generalizability of our findings. There are multiple copper ionophores and cuproptosis inducers available, and whether their mechanisms of inducing cuproptosis and the inhibitory effects of XBP1s are consistent remains uncertain. Comparative studies across different inducers are needed to elucidate the broader implications of copper-mediated cell death and its regulation by XBP1s. Second, while our mouse xenograft models have provided valuable insights into the regulatory axis of XBP1s-MGRN1-LIPT1 in LUAD, they may not fully recapitulate the complexity and heterogeneity of human tumors. Incorporating patient-derived xenografts (PDX) or organoid models in future research would provide a more clinically relevant platform to validate our findings. Additionally, the dual roles of both copper and the UPR remain underexplored in this study. Excessive copper and overactivation of the UPR can ultimately lead to cell death, which reflects the dynamic and heterogeneous nature of copper metabolism and its relationship with UPR activation. How to define the optimal threshold for therapeutic intervention remains an unresolved challenge.

## Conclusions

Our study provides a comprehensive understanding of the interplay between copper accumulation and LUAD progression, identifying XBP1s as a pivotal regulator of the cuproptosis pathway. We demonstrate that elevated copper levels in advanced LUAD tumors activate XBP1s-mediated SEs, leading to increased MGRN1 transcription. This enhances LIPT1 ubiquitination and degradation, promoting glycolysis while inhibiting cuproptosis. Our findings underscore the therapeutic potential of copper ionophores combined with strategies targeting XBP1s-related SEs, offering a novel avenue for LUAD treatment. Furthermore, XBP1s shows promise as a biomarker for predicting LUAD responsiveness to copper-based therapies. These insights deepen our understanding of copper-mediated tumor biology and lay a foundation for future clinical applications, including the development of more effective combination therapies.

## Supplementary information

Supplementary figures

aj-checklist

Original Western blot

## Data Availability

The data that support the findings of this study are available upon request from the corresponding author.
