# Supplementary material for: Endoplasmic reticulum stress related super-enhancers suppress cuproptosis via glycolysis reprogramming in lung adenocarcinoma
Source: Cell Death Dis. 2025 Apr 19;16(1):316. doi: 10.1038/s41419-025-07613-0 (PMC12009302; doi:10.1038/s41419-025-07613-0)
Supplement: Supplementary file 1 — Supplementary figures [file 41419_2025_7613_MOESM1_ESM.pdf]

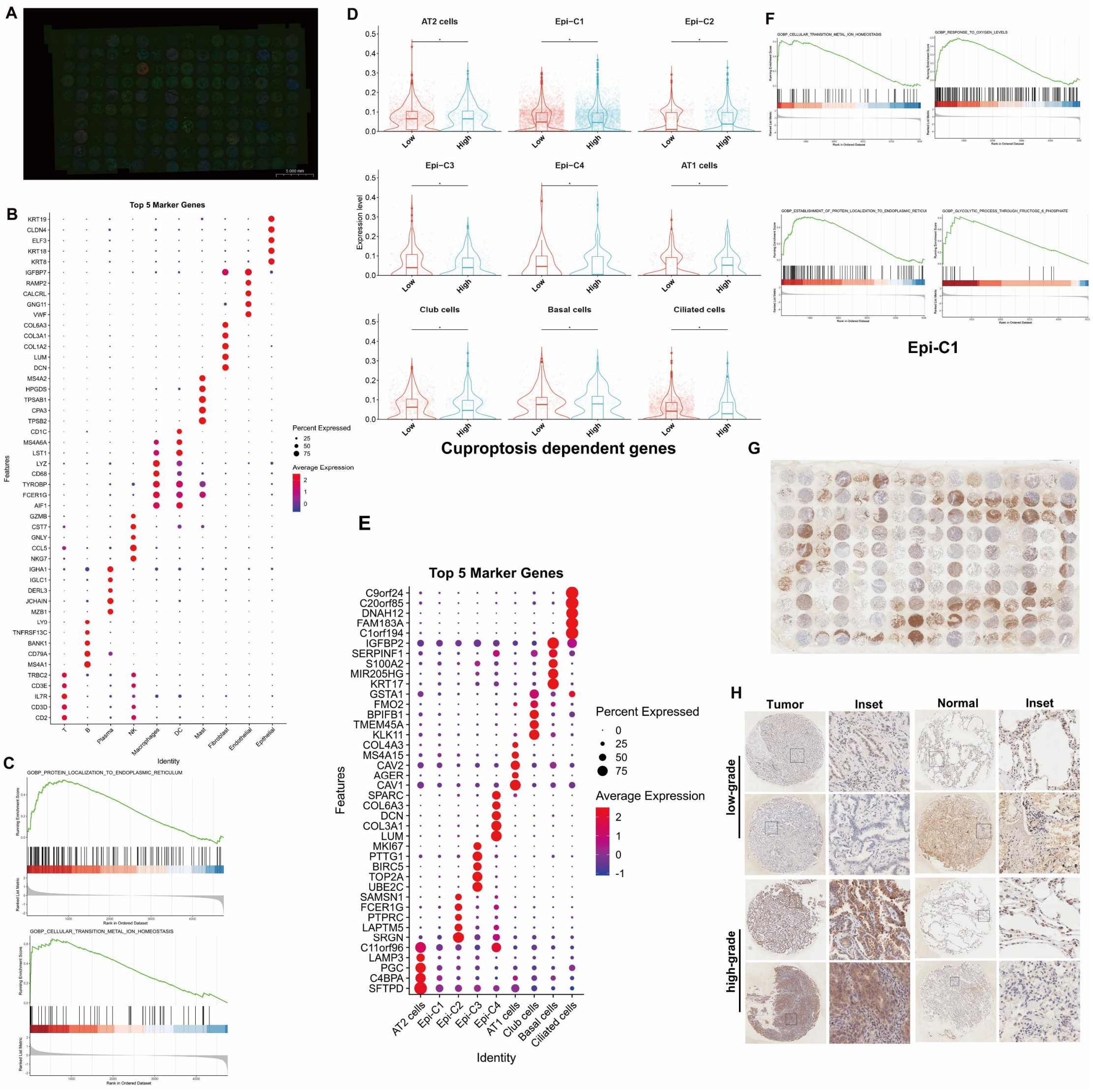

Fig. S1: Glycolysis activation and high expression of XBP1s are accompanied in high-grade LUAD

(A) Overview image of fluorescent staining microarray for DLAT and FDX1 in LUAD tissues. **(B)** Dot plot visualization of single-cell RNA sequencing (scRNA-seq) analysis, identifying marker genes for each cell cluster in LUAD tissues. Top five marker genes are shown for epithelial subclusters and other major cell types, with dot size representing the percentage of cells expressing each gene and color intensity reflecting the average expression level. **(C)** GSEA results indicating significant enrichment of pathways (Protein localization to endoplasmic reticulum and Metal ion

homeostasis) in high-grade LUAD tissues compared to low-grade samples. These pathways include oxidative phosphorylation and mitochondrial-related metabolic processes, underscoring the metabolic rewiring in aggressive tumors. **(D)** Expression levels of cuproptosis-dependent genes in epithelial subclusters (AT2 cells, Epi-C1, Epi-C2, Epi-C3, Epi-C4, AT1 cells, club cells, basal cells, and ciliated cells) as revealed by scRNA-seq analysis. Epi-C1, Epi-C3 and Epi-C4 demonstrating higher expression levels in low-grade group. **(E)** Dot plot analysis of epithelial subclusters, highlighting the top five marker genes for each group. **(G)** Immunohistochemical (IHC) staining of XBP1s on a LUAD tissue microarray, providing an overview of expression across tumor and normal tissues. **(H)** Representative IHC images from the tissue microarray, illustrating XBP1s expression in tumor tissues (low-grade and high-grade) compared to adjacent normal tissues.

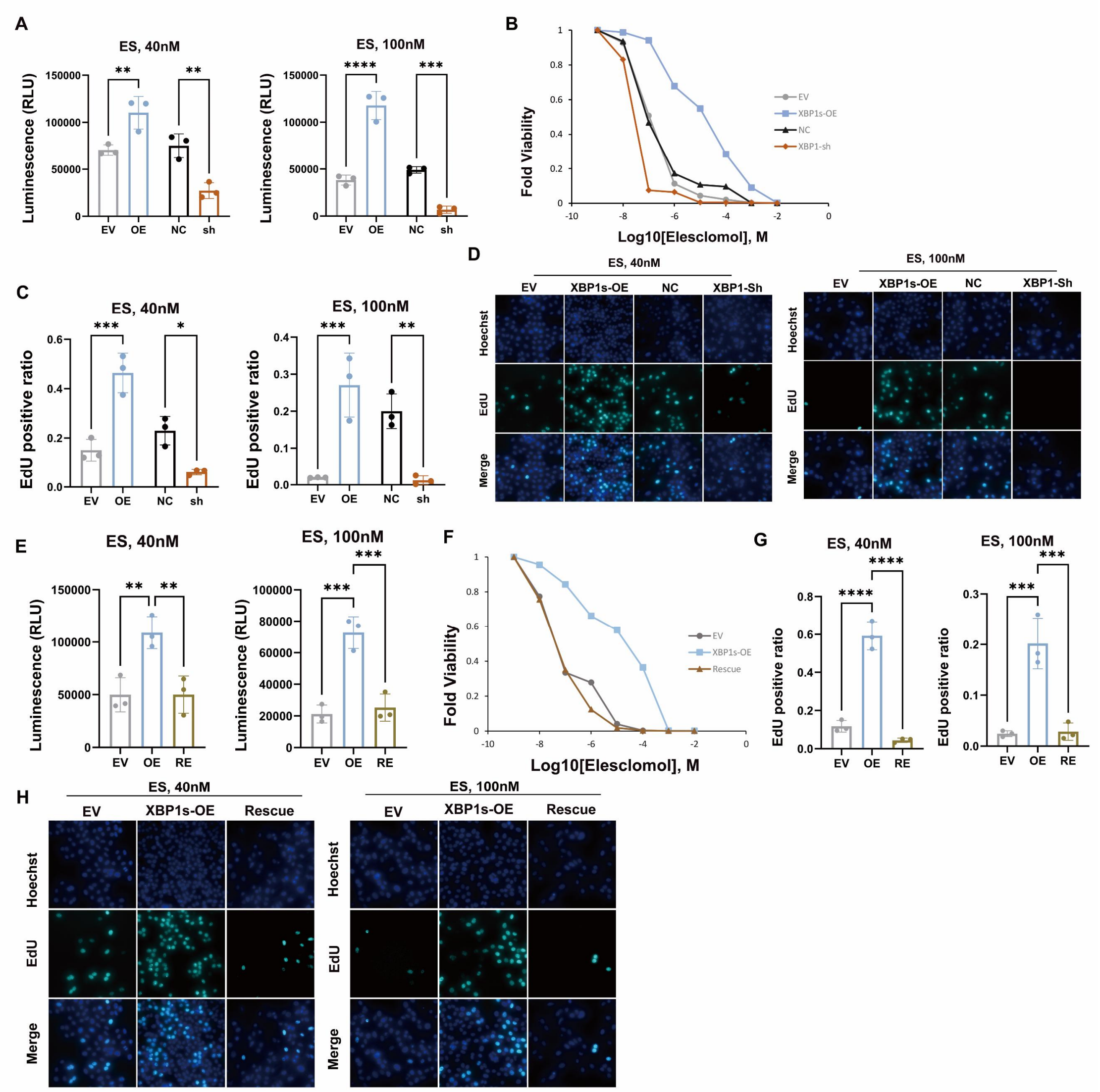

Fig. S2: Functional validation of XBP1s and LIPT1 in PC9 cells under ES treatment.

(A) Cell viability assay showing the effects of EV, OE, NC and sh on cell viability under 40 nM and 100 nM ES treatment. XBP1s-OE significantly enhanced cell viability, while XBP1s-sh reduced it. **(B)** Dose-response curve for cell viability under increasing concentrations of ES. XBP1s-OE increased resistance to cuproptosis, whereas sh sensitized cells to ES-induced cytotoxicity. **(C-D)** EdU incorporation assay under 40 nM and 100 nM ES treatment, quantifying the proliferation rate of PC9 cells with EV, OE, NC, or sh. Representative fluorescence images **(D)** show EdU-positive cells (Indigo, 647nm) and Hoechst-stained nuclei (blue). XBP1s-OE increased the proportion of EdU-positive cells, while XBP1s-sh decreased it significantly. **(E)** Cell viability assay for LIPT1 rescue experiments, showing that reintroduction of LIPT1

(RE) decreased viability in XBP1s-OE cells under 40 nM and 100 nM ES treatment. **(F)** Dose-response curve showing the impact of LIPT1 rescue on ES-induced cytotoxicity. Rescue of LIPT1 expression reversed the enhanced ES resistance conferred by XBP1s-OE. **(G-H)** EdU incorporation assay to evaluate the proliferation rate in LIPT1 rescue experiments under 40 nM and 100 nM ES treatment. Quantitative analysis **(G)** and representative images **(H)** indicate that LIPT1 rescue significantly reduces EdU-positive cells compared to XBP1s-OE alone. Data are presented as mean ± SD (n=3), with statistical significance denoted by *P < 0.05, **P < 0.01, ***P < 0.001 and ****P < 0.0001.

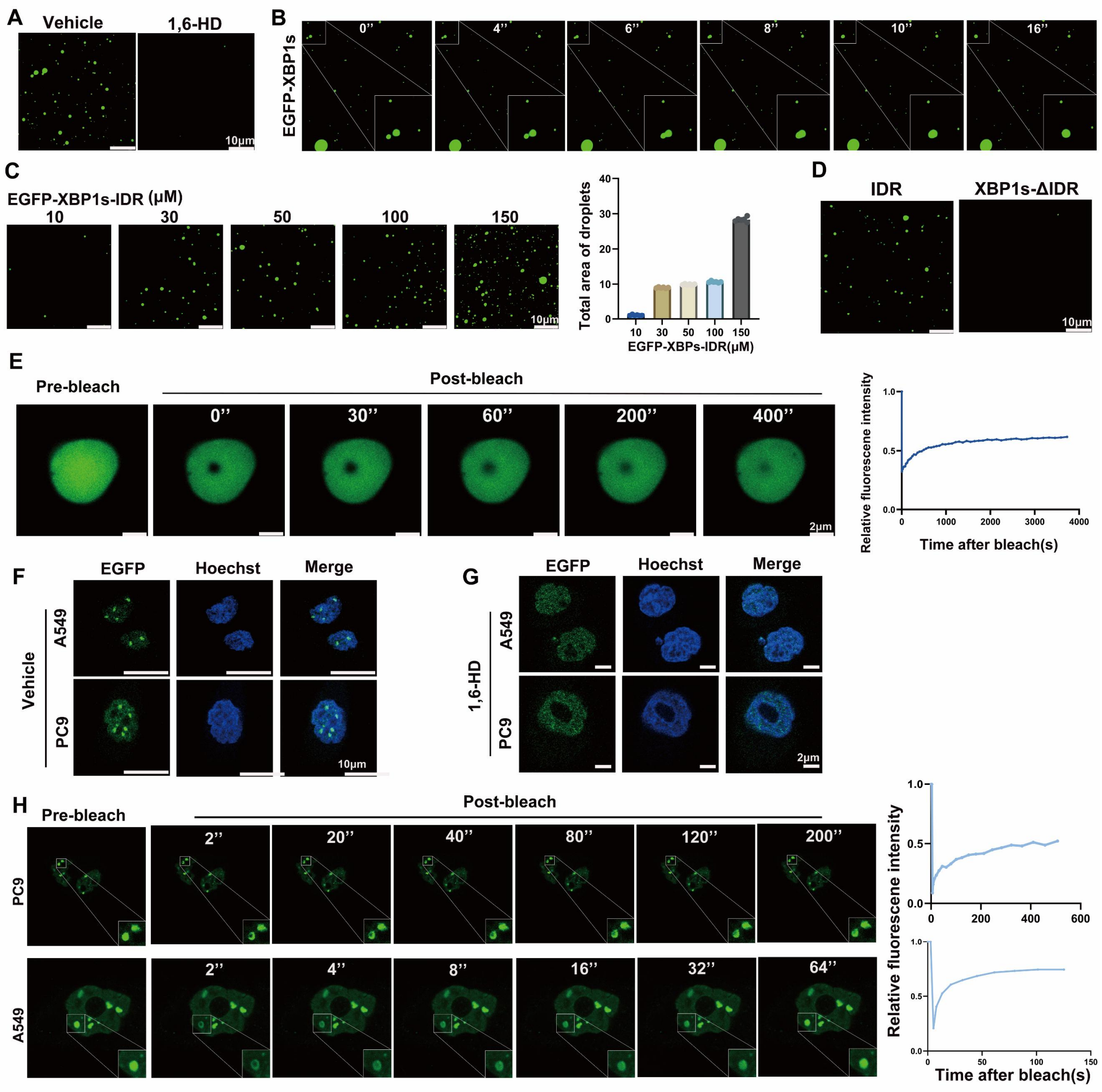

Fig. S3: IDR of XBP1s undergoes phase separation *in vitro* and in cells.

(A) 1,6-HD abolish the EGFP-XBP1s droplets in vitro (488 nm). **(B)** Two EGFP-XBP1s droplets fused over a time course *in vitro*. **(C)** Representative fluorescence

images (488 nm) of EGFP-XBP1s-IDR droplets at different protein concentrations in a buffer containing 125 mM NaCl and 10% PEG-8000. Quantification of droplets area is shown besides. **(D** XBP1s-ΔIDR mutant type failed to form droplets *in vitro*.

(E) FRAP recovery of EGFP-XBP1s-IDR droplets (0-400s). FRAP recovery curves are at right. **(F-G)** Live-cell imaging of A549 and PC9 cells, EGFP-XBP1s-IDR puncta were observed under treatment with buffers, both in the presence and absence of 5% 1,6-HD. Nuclei were distinctly visualized through Hoechst staining. **(H)** FRAP recovery of EGFP-XBP1s-IDR puncta in live A549 and PC9 cells. White squares mark photo-bleached puncta. The fluorescence recovery occurred within 2–64 seconds in A549 cells and 2–200 seconds in PC9 cells, further confirming the dynamic and liquid-like properties of XBP1s-IDR condensates. Quantification of fluorescence recovery is displayed on the right.

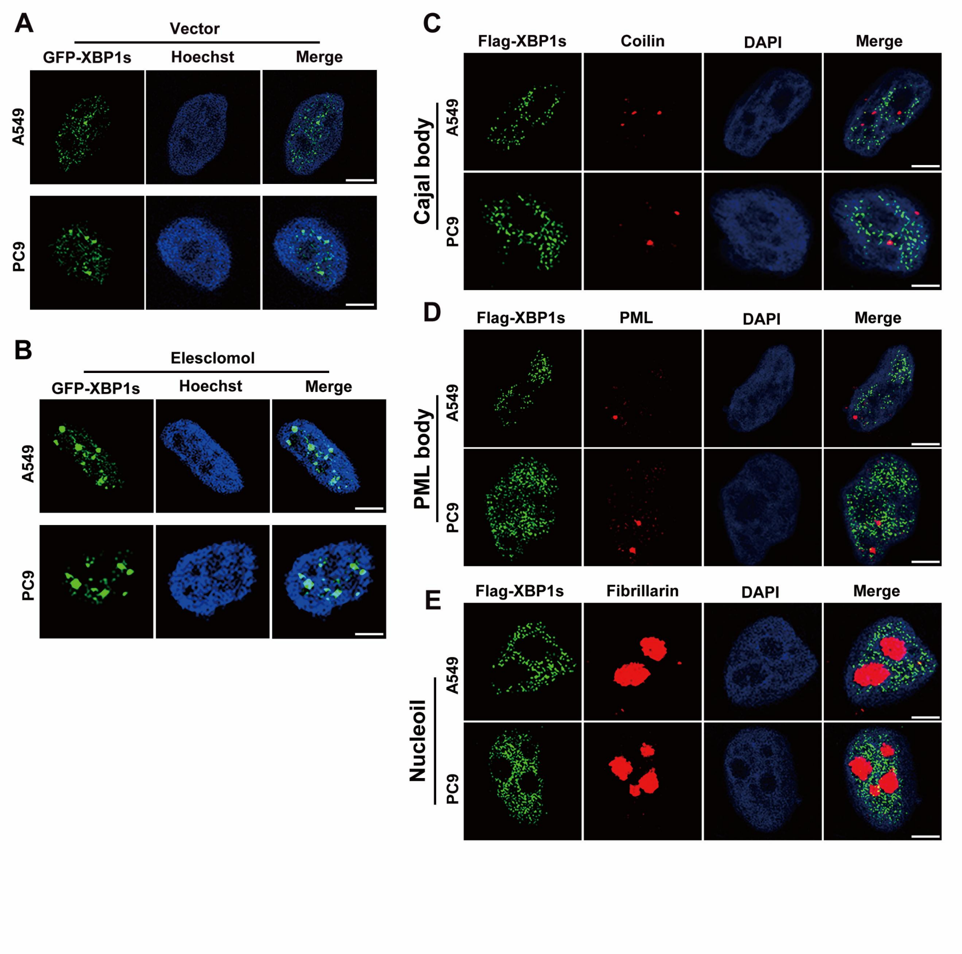

Fig. S4: XBP1s nuclear condensates are promoted by copper and do not co-localize with the PML bodies, Cajal bodies or nucleoli.

(A-B) Treatment of ES (40 nM, 48H) significantly enhances the number and sizes of GFP-XBP1s droplets living cell imaging of LUAD cells.

(C-E) The PML nuclear bodies, Cajal Bodies and nucleoli in A549 and PC9 cells expressing Flag-XBP1s (green) were detected by immunofluorescence staining with antibodies targeting PML, Coilin and Fibrillarin, respectively (red). The results show that Flag-XBP1s does not co-localize with them. Scale bar, 10μm.

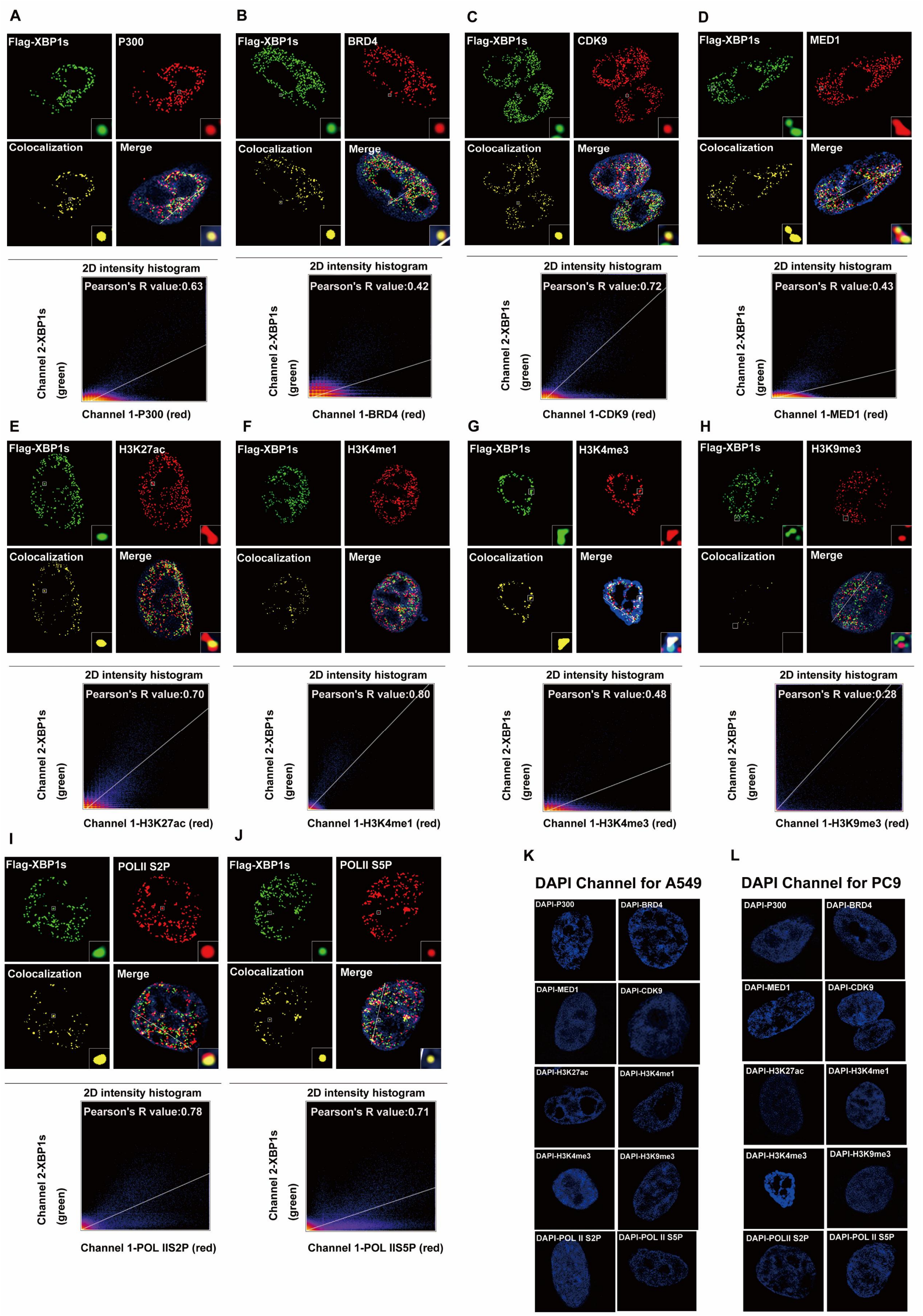

Fig. S5: XBP1s compartmentalizes makers of SEs to nuclear puncta in PC9 cells.

Colocalization of transcriptional coactivators P300 **(A)**, BRD4 **(B),** CDK9 **(C)**, MED1 **(D)** with Flag-XBP1s (green) in nuclear puncta in PC9 cells. Line scans of the

colocalization images are depicted by white arrows with quantification (2D intensity histograms) shown to the right, highlighting Pearson's correlation coefficients (R-values). Areas of colocalization are indicated in yellow. Localization of active histone markers H3K27ac **(E)**, H3K4me1 **(F)** and H3K4me3 **(G)**, repressive histone markers H3K9me3 **(H)** and active RNA POI II S2P **(I)** and S5P **(J)** (red)with Flag-XBP1s (green) in PC9 cells, which suggested that the condensates formed by XBP1s are associated with the transcriptional activation of target genes. Line scans of the colocalization images are depicted by white arrows with quantification (2D intensity histograms) shown to the right and below, highlighting Pearson's correlation coefficients (R-values). Areas of colocalization are indicated in yellow. All DAPI staining in Fig. 5 and Fig. S5 is presented in **(K-L)**, serving as nuclear counterstains to demonstrate the localization of the indicated markers.

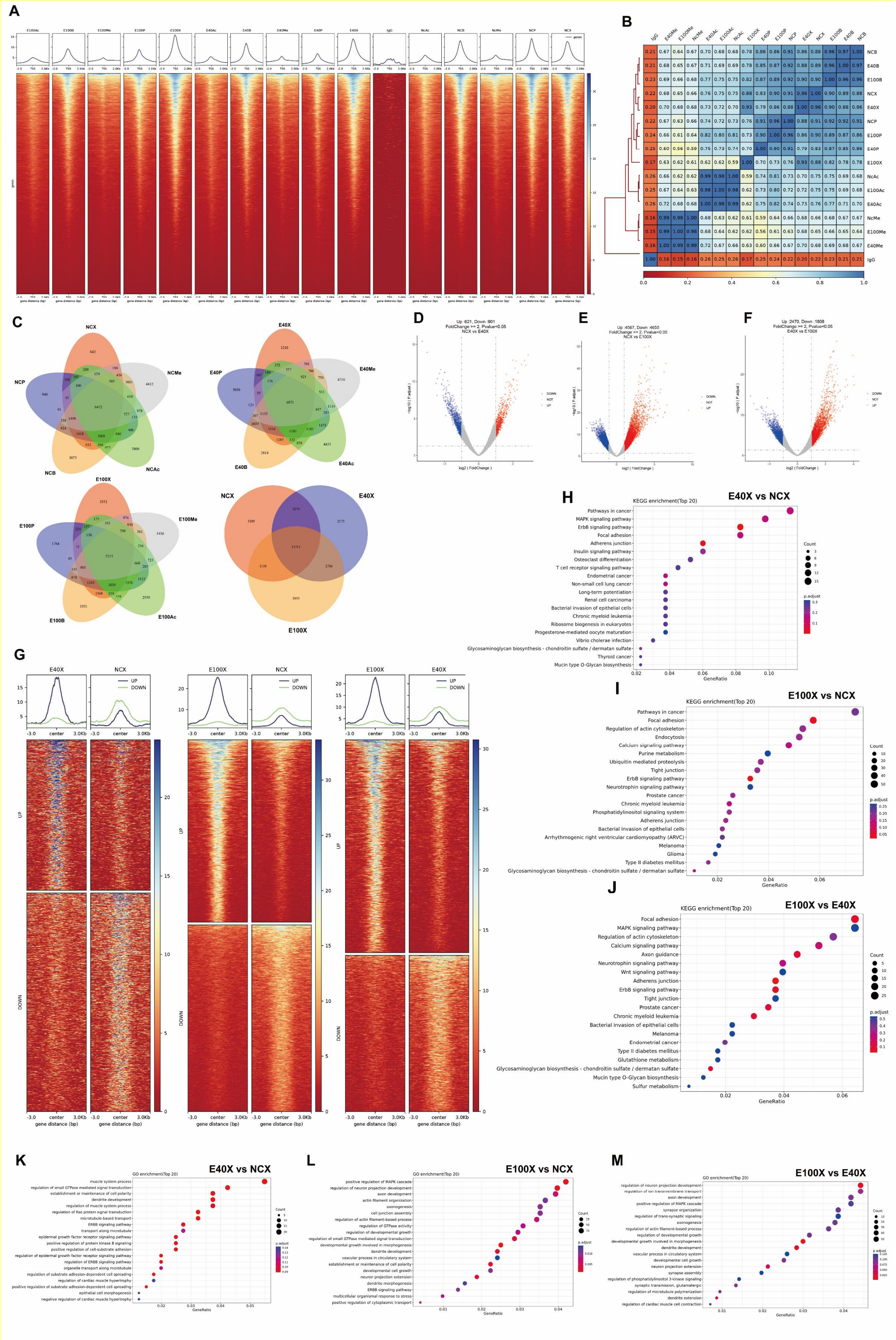

Fig. S6: Comprehensive analysis of XBPs and coactivators via CUT&Tag-sequencing under varying ES concentration conditions.

(A) Heatmap of normalized gene expression profiles across different experimental

conditions (NC: negative control, E40: 40nM elescolmol, E100: 100nM elescolmol, X: XBP1s, P: P300, B: BRD4, Me: MED1, Ac: H3K27ac). Each column represents a sample, and each row represents a gene. The density plots on top indicate the distribution of gene expression levels for each condition. **(B)** Correlation heatmap showing pairwise Pearson correlation coefficients between samples. High correlation coefficients indicate similar gene expression profiles, with hierarchical clustering of samples based on these similarities. **(C)** Venn diagrams illustrating the overlap of differentially expressed genes (DEGs) identified in pairwise comparisons. Overlapping regions represent genes commonly regulated across different conditions. **(D-F)** Volcano plots of DEGs in pairwise comparisons: (D) E40X vs. NCX, (E) E100X vs. NCX, and (F) E100X vs. E40X. Red dots indicate upregulated genes, blue dots indicate downregulated genes, and the horizontal dashed line marks the significance threshold ($p < 0.05$). **(G)** Heatmaps of top differentially expressed genes in specific pairwise comparisons: (left) E40X vs. NCX, (middle) E100X vs. NCX, and (right) E100X vs. E40X. The density plots on top show the distribution of gene expression levels, and the heatmaps display the clustering of samples based on gene expression profiles. **(H-J)** KEGG pathway enrichment analysis of DEGs for: **(H)** E40X vs. NCX, **(I)** E100X vs. NCX, and **(J)** E100X vs. E40X. The x-axis shows the gene ratio, the y-axis lists the enriched KEGG pathways, and the size of the dots corresponds to the number of genes involved. The color gradient indicates the adjusted p-value for pathway enrichment. Notably, the ubiquitination pathway was significantly activated in the E100X group compared to NCX. **(K-M)** Gene Ontology (GO) biological process enrichment analysis of DEGs for: **(K)** E40X vs. NCX, **(L)** E100X vs. NCX, and **(M)** E100X vs. E40X.

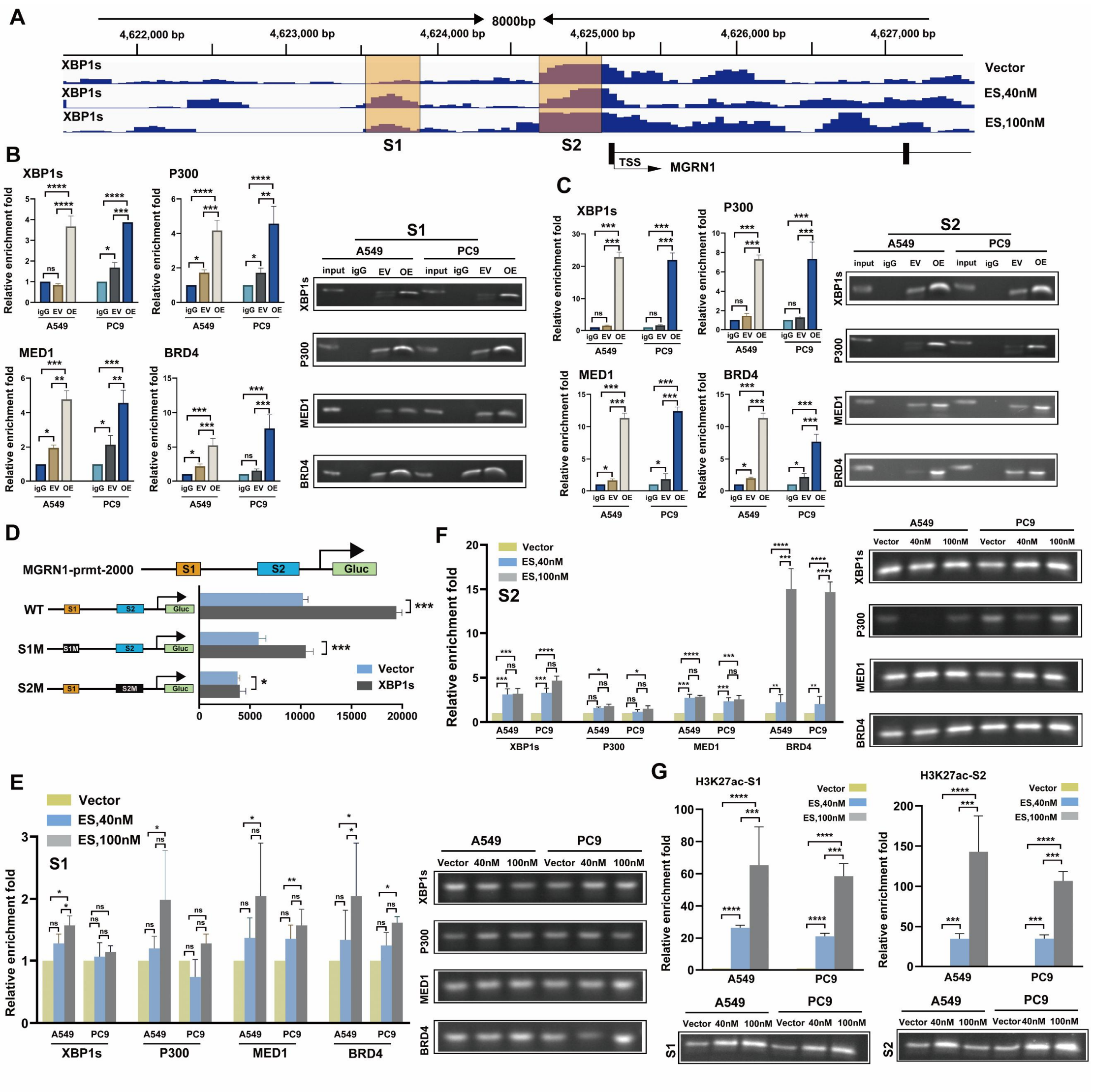

Fig. S7: XBP1s binds to the MGRN1 promoter and activates its expression.

(A) Schematic representation of the XBP1s binding site on the transcription start site (TSS) of the MGRN1 gene locus (chr16:4622826–4624826). Highlighted regions (S1 and S2) indicate potential XBP1s binding sites. **(B-C)** CUT&Tag-qPCR assays were performed to measure the binding of XBP1s and its coactivators (P300, MED1, and BRD4) at the S1 and S2 regions of the MGRN1 promoter in A549 and PC9 cells. Bar graphs represent relative enrichment levels of XBP1s and coactivators normalized to IgG controls. Representative agarose gel images confirm the specific enrichment of proteins at these sites. **(D)** Dual-luciferase reporter assay were used to evaluate the

functional contribution of S1 and S2 binding sites in the MGRN1 promoter. Wild-type (WT) and mutant promoter constructs (S1M: mutated S1 site; S2M: mutated S2 site) were cloned upstream of a luciferase reporter gene. Mutation of the S2 site caused a more pronounced reduction in luciferase activity compared to the S1 site mutation, indicating the dominant role of the S2 site in XBP1s-mediated transcriptional regulation. **(E-F)** Effect of ES treatment on XBP1s and coactivator binding to the S1 and S2 sites as assessed by CUT&Tag-qPCR assays. ES treatment (40nM and 100nM) significantly enhanced the binding of XBP1s and coactivators, particularly BRD4, at the S2 site. Enrichment levels increased in an ES dose-dependent manner. Quantitative data are shown representative agarose gel images. **(G)** Histone modification analysis at the S1 and S2 sites. CUT&Tag-qPCR results showed a marked increase in H3K27ac enrichment at both S1 and S2 sites upon ES treatment (40nm and 100nm). The effect was more pronounced at the S2 site, correlating with its stronger transcriptional regulatory role. All data were analyzed using one-way or two-way ANOVA with post hoc Tukey's test for multiple comparisons. Results are presented as mean ± SD (n=3). Statistical significance: $*P < 0.05$, $**P < 0.01$, $***P < 0.001$, $****P < 0.0001$.

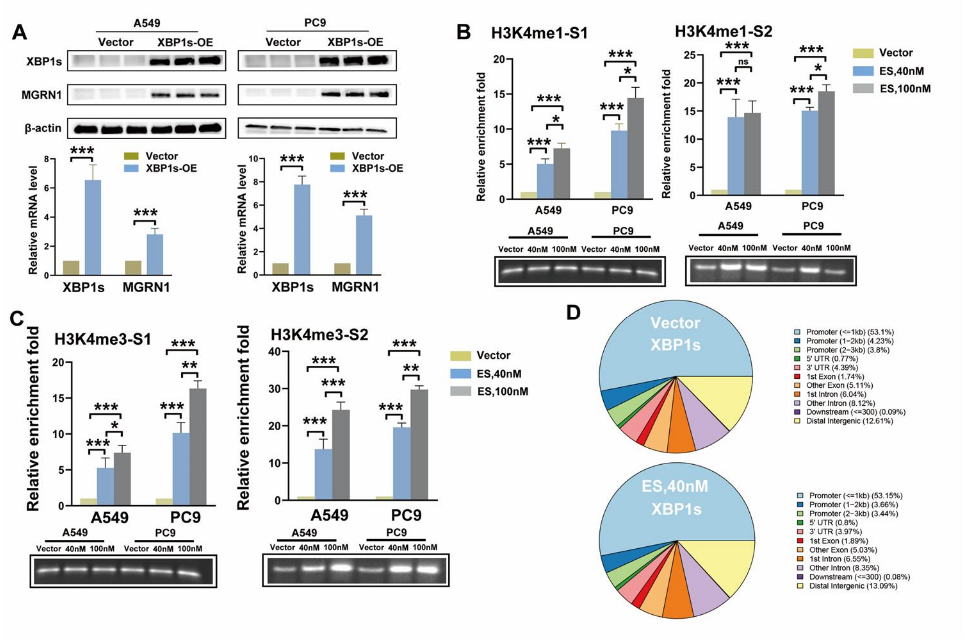

Fig. S8: Effect of ES Treatment on the Enrichment of H3K4me1 and H3K4me3 at the MGRN1 Promoter.

(A) qPCR and Western Blot analysis showed that overexpression of XBP1s (XBP1s-OE) significantly upregulates the mRNA and protein levels of MGRN1 in A549 and PC9 cells. **(B-C)** CUT&Tag-qPCR Analysis of H3K4me1 and H3K4me3 at the S1 and S2 Sites: ES treatment significantly enhances the enrichment levels of H3K4me1 **(B)** and H3K4me3 **(C)** at both the S1 and S2 promoter regions of MGRN1 in a dose-dependent manner. Results are presented as mean ± SD, with one-way ANOVA followed by Tukey's multiple comparisons test for statistical analysis. * $P < 0.05$, ** $P < 0.01$, *** $P < 0.001$, **** $P < 0.0001$ Representative agarose gel images of CUT&Tag-qPCR products are displayed below each graph. **(D)** Pie charts illustrating the proportion of XBP1s binding sites across various genomic regions under vector control or 40nm ES treatment conditions.

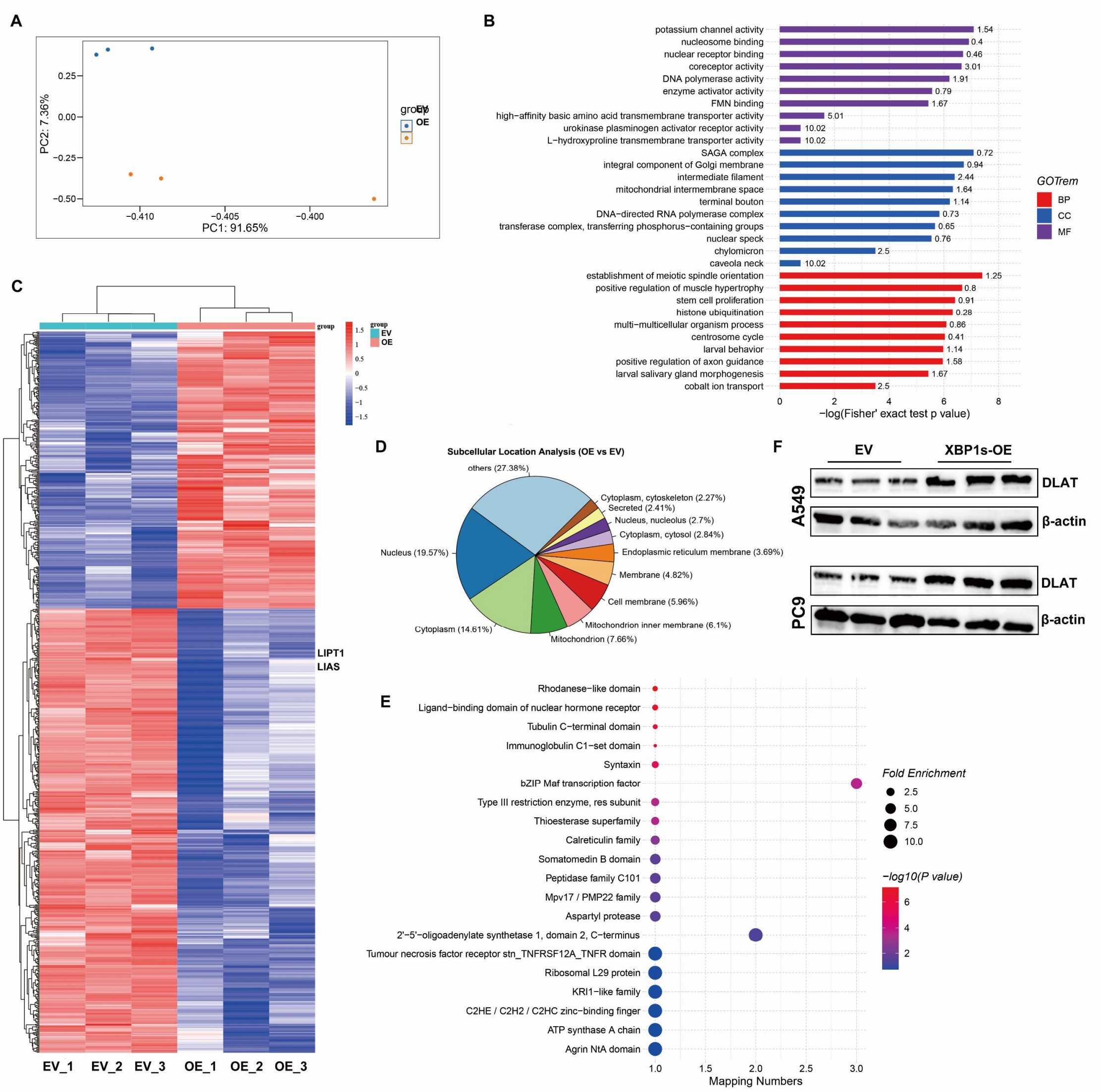

Fig. S9: 4D-DIA proteomics analysis of XBP1s overexpression.

(A) Principal Component Analysis (PCA) plot illustrating distinct clustering of proteomic profiles between XBP1s-overexpressing (OE) and empty vector (EV) groups, indicating significant changes in protein expression upon XBP1s overexpression. **(B)** Gene Ontology (GO) enrichment analysis of differentially expressed proteins (DEPs) between OE and EV groups. GO terms are categorized into biological process (BP), molecular function (MF), and cellular component (CC) domains, with corresponding -log (Fisher exact test p-value) scores. **(C)** Heatmap showing the clustering of DEPs in EV and OE groups. Upregulated and downregulated proteins in the OE group are visualized in red and blue, respectively. Key proteins of cuproptosis such as LIPT1 and LIAS are highlighted. **(D)** Subcellular location analysis of DEPs, showing the distribution of identified proteins across various cellular compartments. Mitochondria-

related proteins constitute 6.1% of the DEPs, emphasizing the impact of XBP1s overexpression on mitochondrial functions. **(E)** Protein domain enrichment analysis of DEPs, highlighting significantly enriched protein domains based on fold enrichment and -log(p-value). **(F)** Western blot analysis validating the increased expression of DLAT in XBP1s-overexpressing (OE) cells compared to empty vector (EV) controls in A549 and PC9 cell lines. β-actin is used as a loading control.

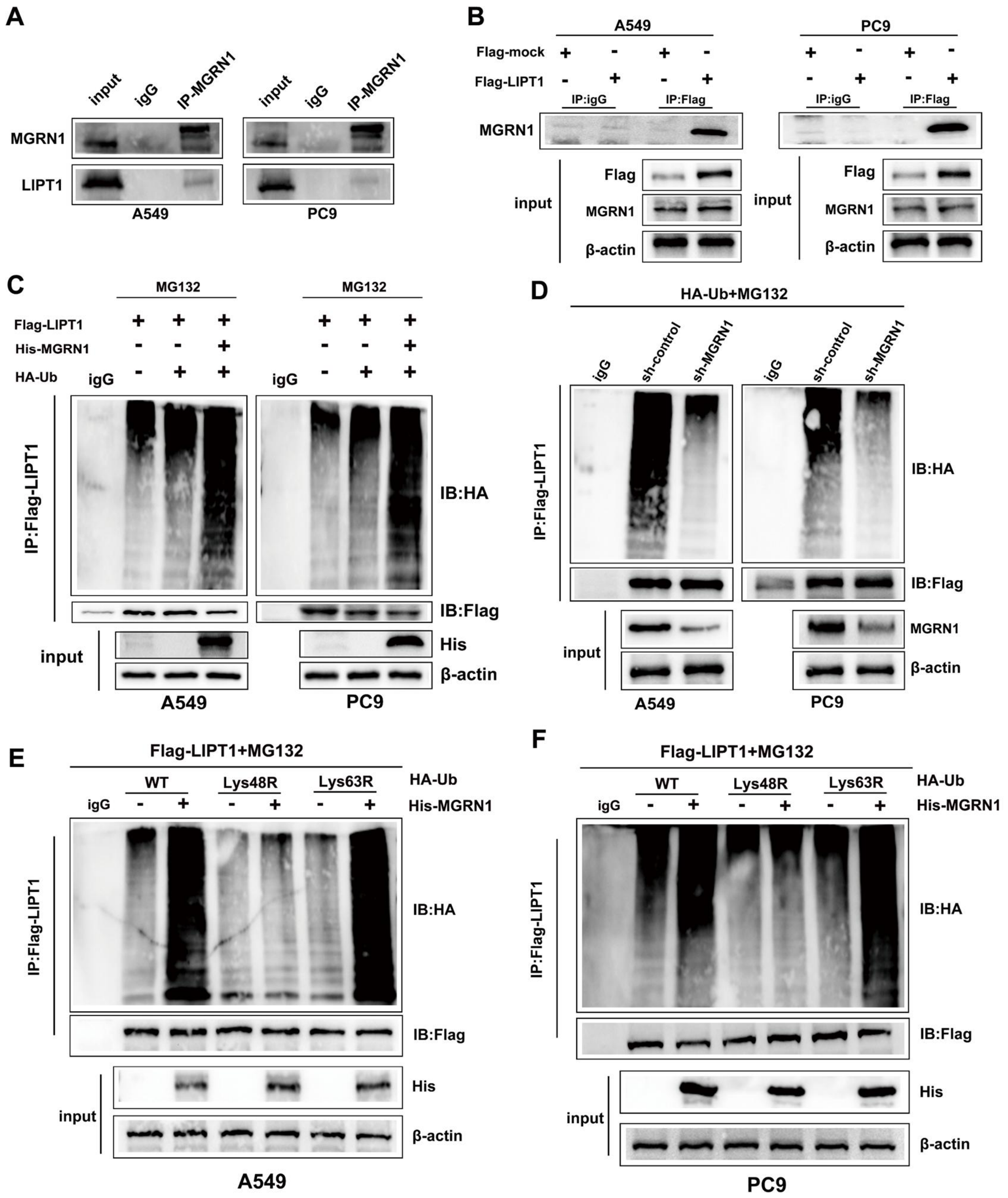

Fig. S10: MGRN1 Promotes the Ubiquitination of LIPT1 via Lys-48.

(A) Co-immunoprecipitation (Co-IP) assays demonstrating the interaction between MGRN1 and LIPT1 in LUAD cells. Anti-MGRN1 antibodies were used to

immunoprecipitate endogenous LIPT1, with IgG as a negative control. (B) Co-IP assays confirming the interaction between exogenous MGRN1 and LIPT1. A549 and PC9 cells were transfected with Flag-LIPT1 or control plasmids, and anti-Flag antibodies were used to immunoprecipitate Flag-LIPT1. Immunoblotting demonstrates the binding of exogenous MGRN1 and LIPT1. **(C)** MGRN1 promotes the ubiquitination of LIPT1. A549 and PC9 cells were transfected with Flag-LIPT1, His-MGRN1, and HA-Ub (ubiquitin), followed by MG132 treatment to inhibit proteasomal degradation. Co-IP assays with anti-Flag antibodies show increased ubiquitinated LIPT1 (IB: HA). **(D)** Knockdown of MGRN1 reduces the ubiquitination of LIPT1. A549 and PC9 cells were transfected with sh-MGRN1 or sh-control along with Flag-LIPT1 and HA-Ub, followed by MG132 treatment. Co-IP with anti-Flag antibodies shows decreased ubiquitinated LIPT1 in MGRN1 knockdown cells. **(E-F)** The Lys48- and Lys63-linked ubiquitin chains may involved in MGRN1-mediated ubiquitination of LIPT1. A549 **(E)** and PC9 **(F)** cells were transfected with WT-ubiquitin, Lys48R (K48R, deficient in forming Lys48-linked chains), or Lys63R (K63R, deficient in forming Lys63-linked chains) mutants, along with Flag-LIPT1 and His-MGRN1. Co-IP assays with anti-Flag antibodies reveal that ubiquitination of LIPT1 is abolished with the K48R mutant, indicating Lys48-linked ubiquitination is predominant. In contrast, the K63R mutant has no significant effect, suggesting Lys63-linked ubiquitination is not critical in this process.

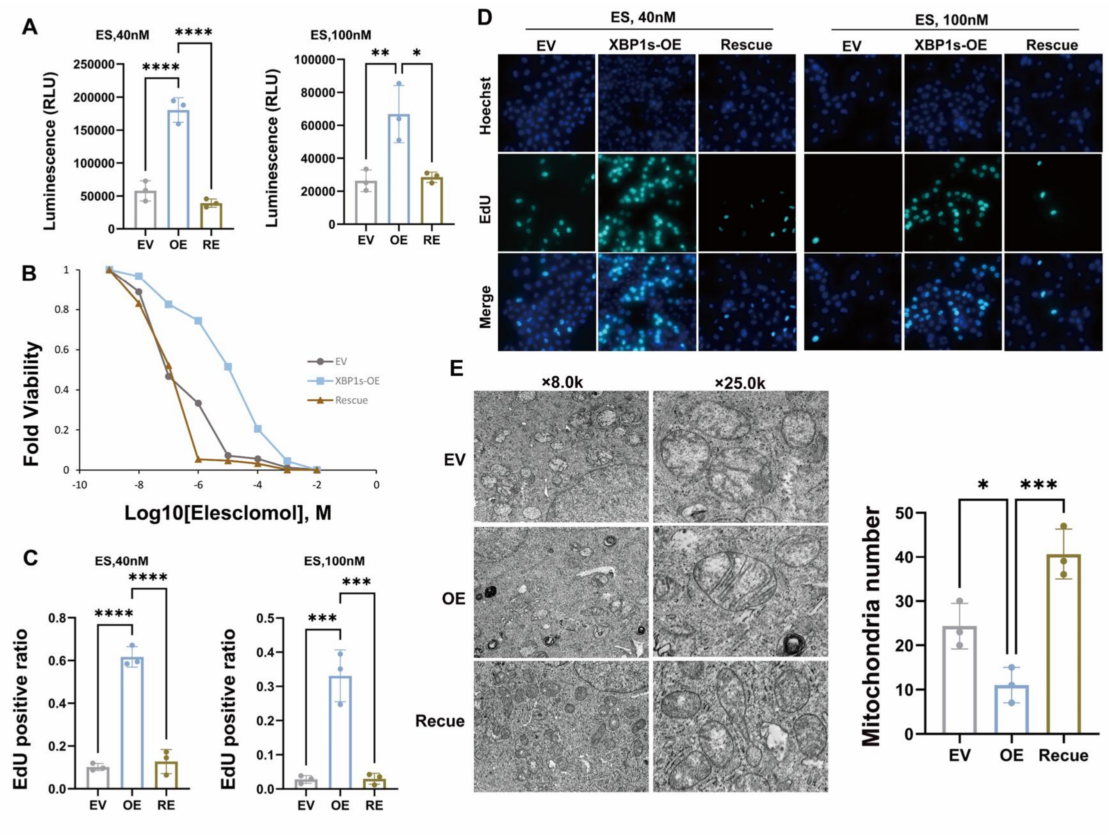

Fig. S11: Overexpression of LIPT1 abrogates the copper death resistance effect of XBP1s in A549 cells.

(A) Cell viability assay for LIPT1 rescue experiments, showing that reintroduction of LIPT1 (RE) decreased viability in XBP1s-OE A549 cells under 40 nM and 100 nM ES treatment. **(B)** Dose-response curve showing the impact of LIPT1 rescue on ES-induced cytotoxicity. Rescue of LIPT1 expression reversed the enhanced ES resistance conferred by XBP1s-OE. **(C-D)** EdU incorporation assay to evaluate the proliferation rate in LIPT1 rescue experiments under 40 nM and 100 nM ES treatment. Quantitative analysis **(C)** and representative images **(D)** indicate that LIPT1 rescue significantly reduces EdU-positive cells compared to XBP1s-OE alone. **(E)** High-resolution TEM image of the sample acquired using a Hitachi HT7700 microscope. The EV and Rescue groups have more mitochondria compared to the OE group, but with abnormal

morphology, including the loss of mitochondrial cristae and mitochondrial swelling. Differences of mitochondrial numbers were detected in the bar chart (n=3). Data are presented as mean ± SD (n=3), with statistical significance denoted by *P < 0.05, **P < 0.01, ***P < 0.001 and ****P < 0.0001.
