# Supplementary material for: Endoplasmic reticulum stress related super-enhancers suppress cuproptosis via glycolysis reprogramming in lung adenocarcinoma
Source: Cell Death Dis. 2025 Apr 19;16(1):316. doi: 10.1038/s41419-025-07613-0 (PMC12009302; doi:10.1038/s41419-025-07613-0)
Supplement: Supplementary file 3 — Original Western blot [file 41419_2025_7613_MOESM3_ESM.pdf]

Fig3.A

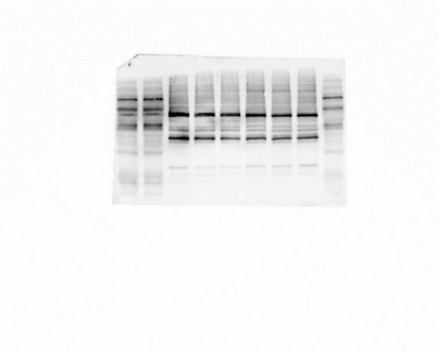

anti-XBP1u (about 35kda)

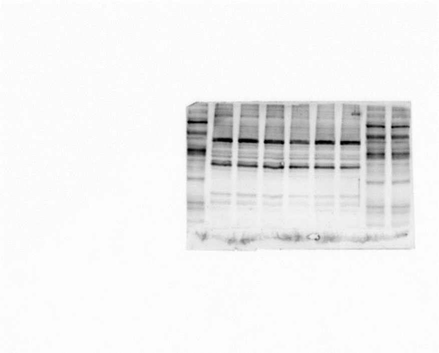

anti-XBP1u

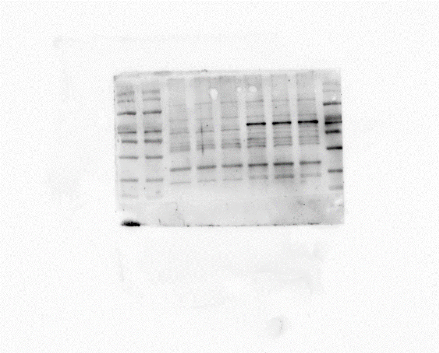

anti-XBP1s (about 55kda)

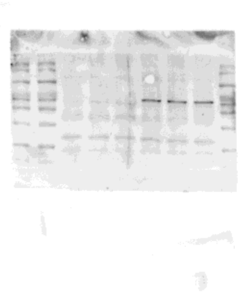

anti-XBP1s

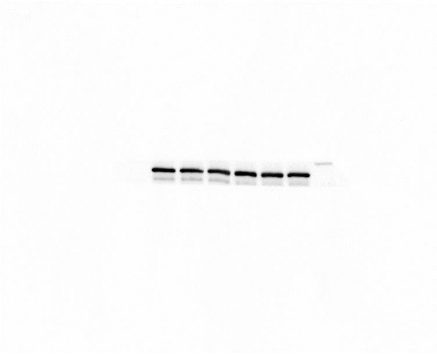

anti-β-actin

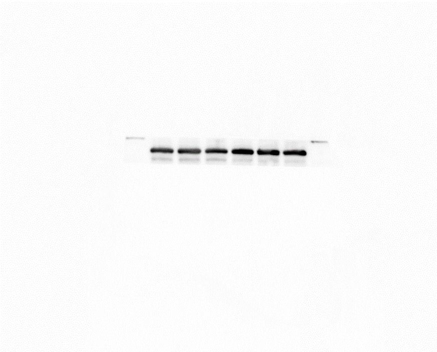

anti-β-actin

Fig7. C-D

anti-LIPT1 (about 42kda)

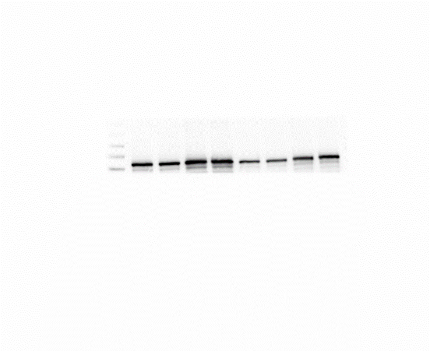

anti-XBP1s

anti-β-actin

anti-LIPT1

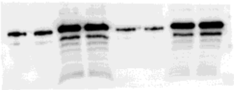

anti-XBP1s

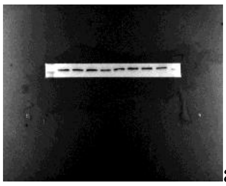

anti-β-actin

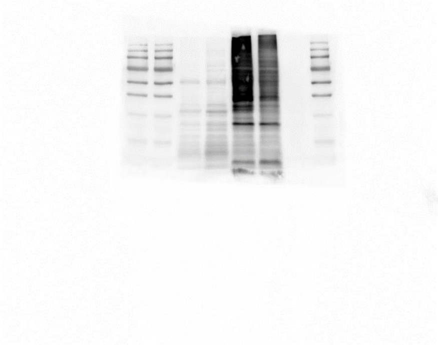

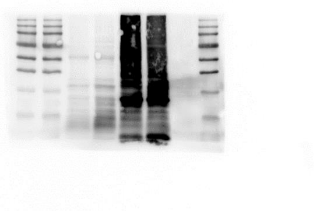

anti-HA

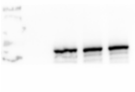

anti-Flag

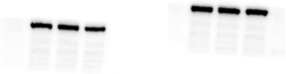

anti-β-actin

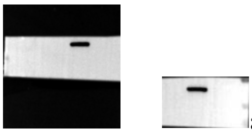

anti-Myc

Fig8. A

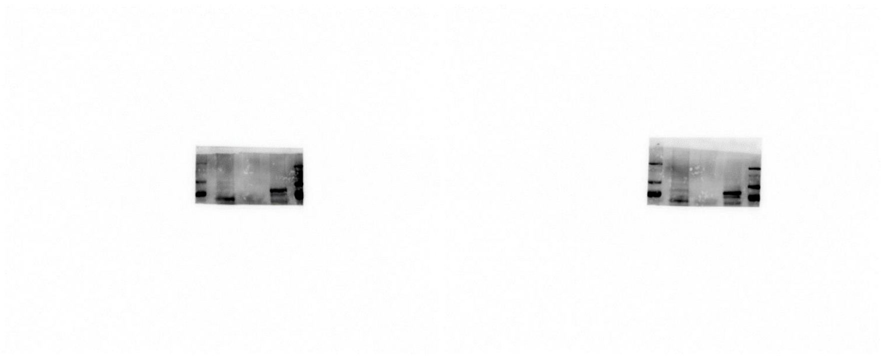

anti-MGRN1

(about 60kda)

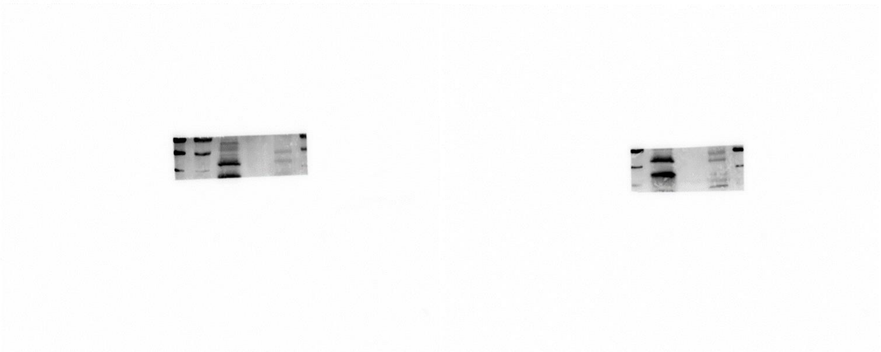

anti-LIPT1

Fig8. B

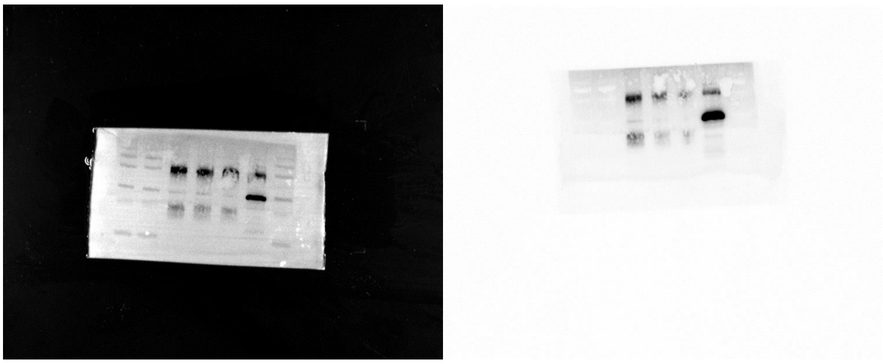

anti-MGRN1

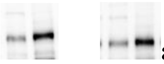

anti-Flag

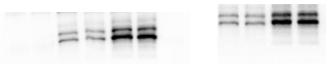

anti-β-actin

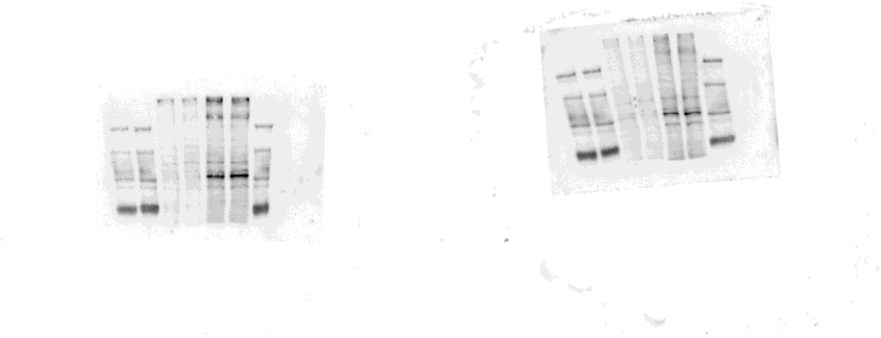

anti-MGRN1

Fig8. C

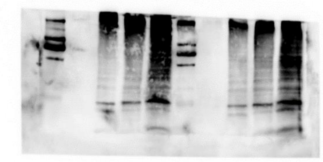

anti-HA

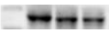

anti-Flag

anti-Flag

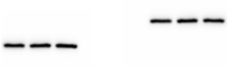

anti-β-actin

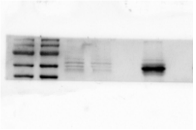

anti-His

Fig8. D

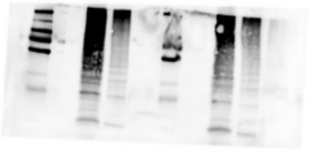

anti-HA

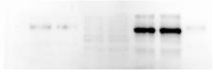

anti-Flag

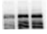

anti-Flag

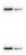

anti-MGRN1

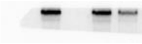

anti-MGN1

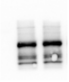

anti-β-actin

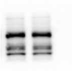

anti-β-actin

Fig8. E

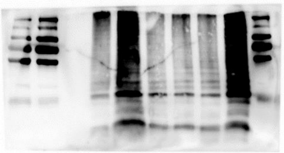

anti-HA

anti-Flag

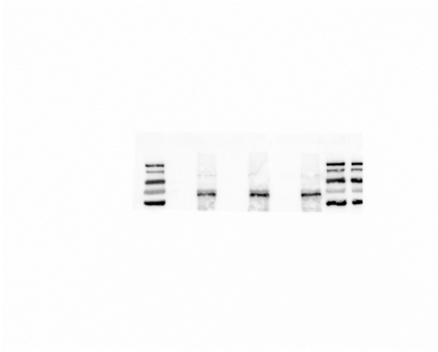

anti-His

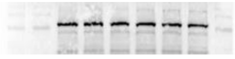

anti-β-actin

Fig8. F

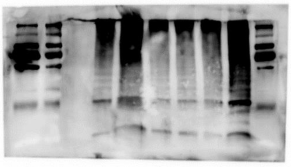

anti-HA

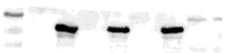

anti-His

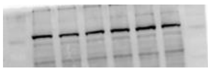

anti-Flag

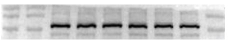

anti-β-actin

FigS8. A

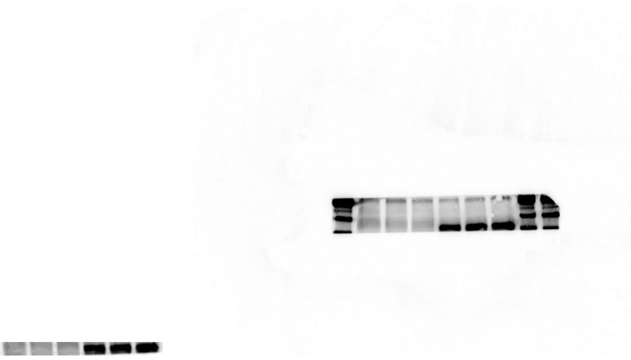

anti-XBP1s

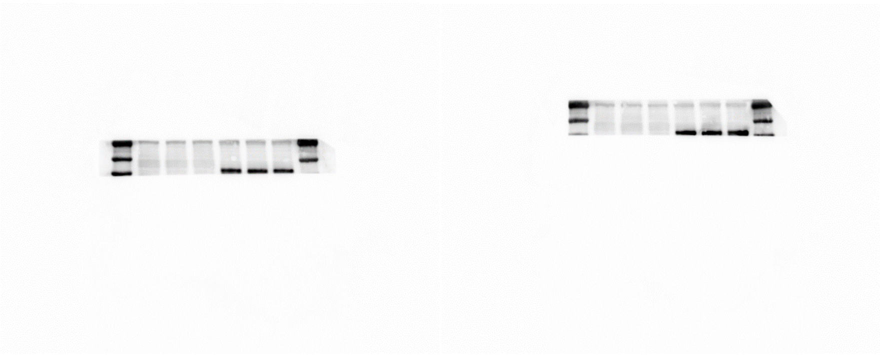

anti-MGRN1

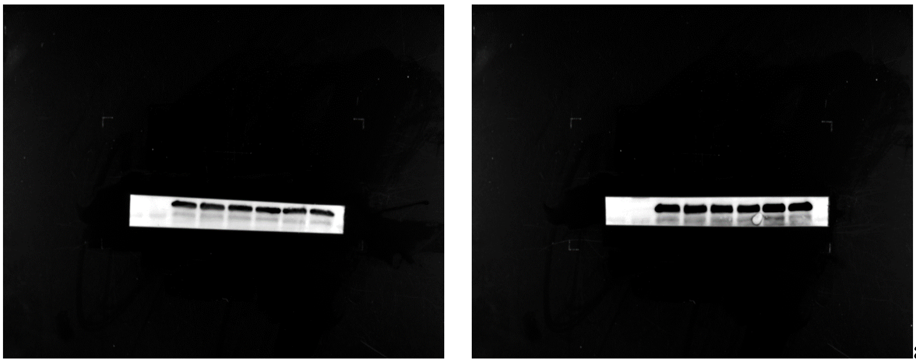

anti-β-actin

FigS9. F

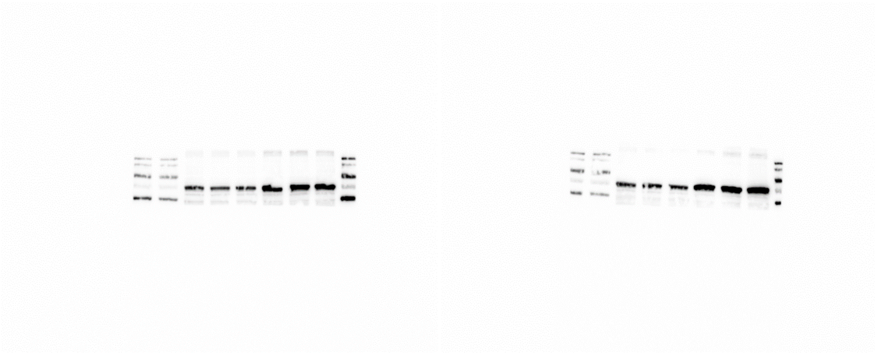

anti-DLAT (about 70kda)

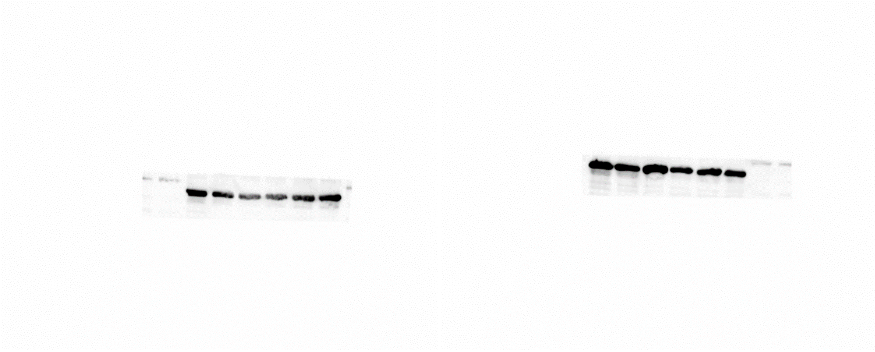

anti-β-actin
